# Marinobazzanan, a Bazzanane-Type Sesquiterpenoid, Suppresses the Cell Motility and Tumorigenesis in Cancer Cells

**DOI:** 10.3390/md21030153

**Published:** 2023-02-25

**Authors:** Sultan Pulat, Prima F. Hillman, Sojeong Kim, Ratnakar N. Asolkar, Haerin Kim, Rui Zhou, İsa Taş, Chathurika D. B. Gamage, Mücahit Varlı, So-Yeon Park, Sung Chul Park, Inho Yang, Jongheon Shin, Dong-Chan Oh, Hangun Kim, Sang-Jip Nam, William Fenical

**Affiliations:** 1 College of Pharmacy and Research Institute of Life and Pharmaceutical Sciences, Sunchon National University, Sunchon 57922, Republic of Korea; 2 Department of Chemistry and Nanoscience, Ewha Womans University, Seoul 03760, Republic of Korea; 3 Graduate School of Industrial Pharmaceutical Sciences, Ewha Womans University, Seoul 03760, Republic of Korea; 4 Center for Marine Biotechnology and Biomedicine, Scripps Institution of Oceanography, University of California-San Diego, La Jolla, CA 92093-0204, USA; 5 Natural Products Research Institute, College of Pharmacy, Seoul National University, San 56-1, Sillim, Gwanak, Seoul 08826, Republic of Korea; 6 Department of Convergence Study on the Ocean Science and Technology, Korea Maritime and Ocean University, Busan 49112, Republic of Korea

**Keywords:** marinobazzanan, bazzanane-type sesquiterpenoid, *Acremonium* sp., cytotoxicity, anticancer

## Abstract

Marinobazzanan (**1**), a new bazzanane-type sesquiterpenoid, was isolated from a marine-derived fungus belonging to the genus *Acremonium*. The chemical structure of **1** was elucidated using NMR and mass spectroscopic data, while the relative configurations were established through the analysis of NOESY data. The absolute configurations of **1** were determined by the modified Mosher’s method as well as vibrational circular dichroism (VCD) spectra calculation and it was determined as 6*R*, 7*R*, 9*R*, and 10*R*. It was found that compound **1** was not cytotoxic to human cancer cells, including A549 (lung cancer), AGS (gastric cancer), and Caco-2 (colorectal cancer) below the concentration of 25 μM. However, compound **1** was shown to significantly decrease cancer-cell migration and invasion and soft-agar colony-formation ability at concentrations ranging from 1 to 5 μM by downregulating the expression level of KITENIN and upregulating the expression level of KAI1. Compound **1** suppressed β-catenin-mediated TOPFLASH activity and its downstream targets in AGS, A549, and Caco-2 and slightly suppressed the Notch signal pathway in three cancer cells. Furthermore, **1** also reduced the number of metastatic nodules in an intraperitoneal xenograft mouse model.

## 1. Introduction

There were an estimated 19.3 million new cancer cases in 2020 and almost 10 million cancer deaths worldwide [1]. Lung cancer is the leading cause of cancer death, with colorectal cancer being the second. Further, gastric cancer is the fourth most common cause of cancer death globally [2]. The World Health Organization estimates that there will be 28.4 million cancer cases worldwide by 2040 [1].

Metastasis refers to secondary tumors which develop in a different part of the body compared to the original cancer. Epithelial-to-mesenchymal transition (EMT) is one of the reasons for metastasis. The invasiveness and metastatic potential of solid tumors increase with EMT. The EMT transcription factors, Snail, Slug, and Twist, are promoted by EMT processes [3]. The Wnt/β-catenin pathway, which promotes the stemness, deterioration, and metastasis of cancer cells, could be another contributing factor to cancer. β-catenin promotes the transcription of a wide range of oncogenes, including c-Myc and CyclinD-1 in the nucleus [4]. Several human malignancies exhibit an increased ability to invade and metastasize when KAI1 (a metastatic suppressor gene) expression is suppressed and KITENIN is promoted. Cancer invasion and metastasis are also mediated through the KITENIN/AP-1 axis, another signal transduction component [5]. The Notch signaling pathway is found in a wide range of solid tumors and can be responsible for both cell proliferation and metastasis, including EMT. The Notch ligand is a single transmembrane protein, and activation of Notch occurs when it binds to the Notch ligand of neighboring cells. The transcriptional targets of HES genes can be also regulated by Notch [6]. Therefore, suppressing EMT, the Wnt/β-catenin pathway, KITENIN/AP-1 axis, and the Notch signal pathway has become an important goal for developing anticancer therapeutics.

The biological and geochemical roles of marine fungi have attracted the attention of researchers in many scientific communities [7,8]. Marine fungi and marine-derived fungi are regarded as prolific sources of natural products with unique chemical structures and diverse biological activities [9,10,11]. Therefore, many research groups have focused their attention on culturing marine fungi to discover novel natural products [12,13,14]. In particular, the marine-derived genus *Acremonium* has been studied intensively as a proficient producer of natural products with a wide range of bioactivities [15,16]. Previously, a cyclic pentadepsipeptide, acremonamide, with wound-healing properties has been isolated from the *Acremonium* strain CNQ-049 [17].

**Figure 1:**
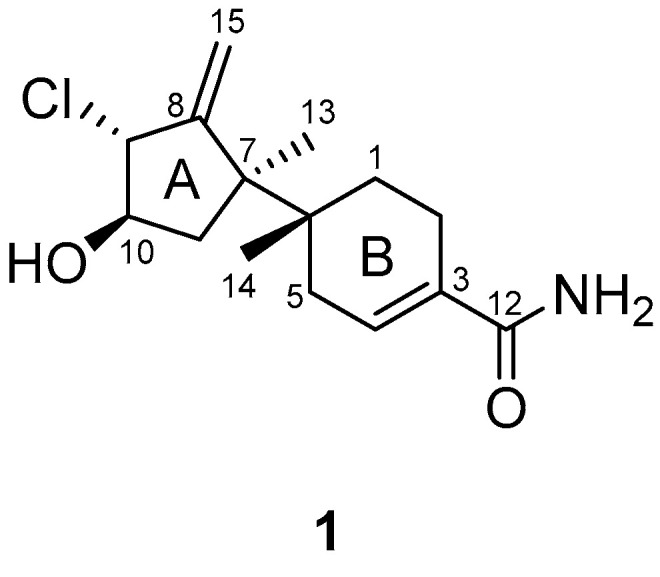
Chemical structure of marinobazzanan (**1**).

Thus, as part of continuing efforts to investigate the chemical components of the *Acremonium* strain CNQ-049, derived from marine sediments collected off the coast of Southern California, we isolated a new bazzanane-type sesquiterpenoid: marinobazzanan (**1**). Although the cancer-cell cytotoxicity of sesquiterpene lactones and their applications for developing anticancer agents have been extensively explored [18], little is known about the bioactivities of bazzanane-type sesquiterpenoids. Hence, the isolation, structural elucidation, and anti-cancer activities of compound **1** (Figure 1) are examined herein.

## 2. Results and Discussion

Marinobazzanan (**1**) was obtained as a pale-yellow amorphous powder. The molecular formula of **1** was determined as C_15_H_22_^35^ClNO_2_ based on a protonated adduct at *m/z* 284.1415 [M + H]^+^ (calculated for C_15_H_23_^35^ClNO_2_, 284.1417) in a high-resolution electrospray ionization mass spectrum (HR-ESI-MS), which indicated five degrees of unsaturation. The ^1^H NMR spectrum of **1** displayed one olefinic (*δ*_H_ 6.66), two exo-methylene (*δ*_H_ 5.44, 5.21), eight methylene (*δ*_H_ 1.54, 1.65, 1.79, 1.87, 2.05, 2.21, 2.21, 2.39), two methine (*δ*_H_ 3.86, 4.29), and two methyl singlet (*δ*_H_ 1.20, 0.92) hydrogens. The ^13^C and HSQC NMR spectroscopic data revealed one carbonyl (*δ*_C_ 172.2), four quaternary (*δ*_C_ 153.7, 131.3, 48.5, 36.8), three methine (*δ*_C_ 134.0, 75.6, 69.2), five methylene (*δ*_C_ 111.9, 40.5, 33.0, 27.4, 21.8), and two methyl singlets (*δ*_C_ 24.5, 17.1) carbons (Table 1, Figures S1 and S2). The molecular formula and the HSQC NMR spectroscopic data of **1** suggested that compound **1** possessed a bicyclic ring system.

**Figure 2:**
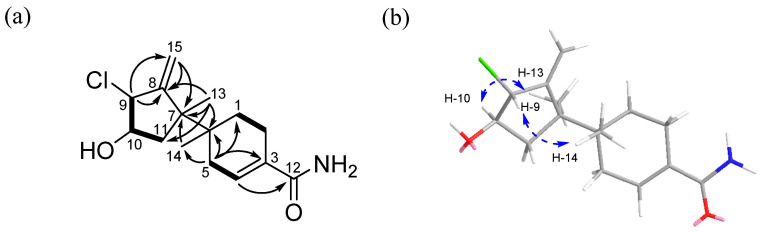
Key COSY, HMBC (**a**) and NOESY (**b**) NMR correlations of marinobazzanan (**1**).

The interpretation of 2D NMR spectra (Figures S3–S5) allowed the construction of a bazzanane-type sesquiterpenoid framework for **1**. The COSY NMR crosspeaks [H-9 (*δ*_H_ 4.29)/H-10 (*δ*_H_ 3.86)/H-11 (*δ*_H_ 1.87, 1.79)] and the long-range HMBC correlations from H-9 (*δ*_H_ 4.29) to C-8 (*δ*_C_ 153.7), C-15 (*δ*_C_ 111.9), from exo-methylene H-15 (*δ*_H_ 5.44, 5.21) to C-7 (*δ*_C_ 48.5), C-8 (*δ*_C_ 153.7), and from the methyl singlet H-13 (*δ*_H_ 1.20) to C-7 (*δ*_C_ 48.5), C-8 (*δ*_C_ 153.7), and C-11 (*δ*_C_ 40.5) allowed the construction of the five-membered ring (ring A) of bazzanane. The six-membered ring (ring B) was established by analyzing the COSY crosspeaks [H-1 (*δ*_H_ 1.65, 1.54)/H-2 (*δ*_H_ 2.39, 2.21) and H-4 (*δ*_H_ 6.66)/H-5 (*δ*_H_ 2.21, 2.05)], along with the long-range HMBC correlations from H-5 (*δ*_H_ 2.21, 2.05) to C-1 (*δ*_C_ 27.4), C-3 (*δ*_C_ 131.3), C-4 (*δ*_C_ 134.0), C-6 (*δ*_C_ 36.8), and C-14 (*δ*_C_ 17.1). In addition, the HMBC NMR correlations from H-4 (*δ*_H_ 6.66) to the carbonyl carbon C-12 (*δ*_C_ 172.2) allowed the connection of the amide group to ring B. Meanwhile, the connection of C-6/C-7 was secured by observing the HMBC correlations from H-13 (*δ*_H_ 1.20) to C-6 (*δ*_C_ 36.8), C-7 (*δ*_C_ 48.5) and from H-14 (*δ*_H_ 0.92) to the same two carbons. Furthermore, the attachment of a hydroxy group at C-10 was assigned through the presence of the carbon chemical shifts at *δ*_C_ 75.6. In addition, a chlorine atom at C-9 was established by considering the isotope ratio (3:1) of the two protonated adduct [M + H]^+^ and [M + H+2]^+^ in the low-resolution electrospray ionization mass spectrum (LR-ESI-MS) and the chemical shift at *δ*_C_ 69.2 in the chlorinated methine, which completed the planar structure of **1**, as shown in Figure 2.

The relative stereochemistry of **1** was determined by analysis of the NOESY NMR spectroscopic data. A NOESY correlation (Figure S6) between H-9 (*δ*_H_ 4.29) and H-14 (*δ*_H_ 0.92) indicated that these protons should be located on the same face of the molecule. Meanwhile, a NOESY crosspeak between H-10 (*δ*_H_ 3.86) and H-13 (*δ*_H_ 1.20) as well as its coupling constant (^3^*J*_H−H_ = 10.0 Hz) established their *syn* relationship. Therefore, the relative configuration of **1** was assigned as 6S*, 7S*, 9S*, and 10S* (Figure 2b).

**Figure 3:**
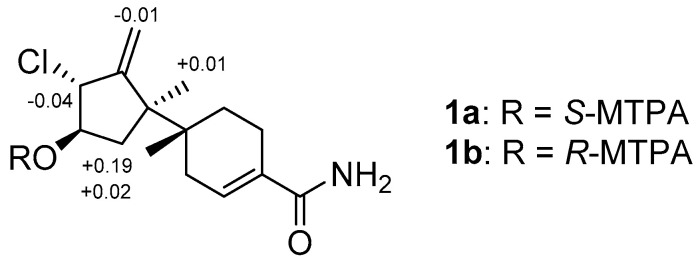
Δ*δ*(S−R) values for the MTPA esters of **1**.

**Figure 4:**
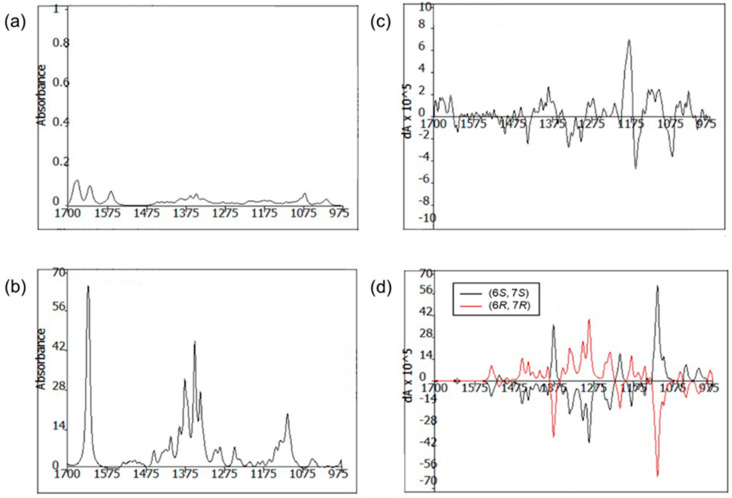
Comparison of the experimental IR (**a**) and VCD (**c**) spectra with the calculated IR (**b**) and VCD (**d**) spectra of structure **1**.

The absolute configuration of **1** was established using the modified Mosher’s method [19,20], combined with a comparison between the measured and calculated vibrational circular dichroism (VCD) spectra [21]. First, esterification of **1** with (*R*)- and (*S*)-MTPA-Cl (*α*-methoxy-*α*-(trifluoromethyl) phenylacetyl chloride) yielded the (*S*)- and (*R*)-MTPA esters of **1** (**1a** and **1b**, respectively). Analysis of the ^1^H NMR (Figures S7 and S8) Δ*δ*_(*S*−*R*)_ values revealed a consistent sign distribution, thus verifying the *R* configuration at C-10 (Figure 3). Therefore, the absolute stereochemistry of the three chiral centers of ring A in **1** was determined as 7*R*, 9*R*, and 10*R*. The absolute configurations of C-6 were also confirmed by comparing the experimental and calculated VCD spectra. The VCD spectra were calculated using density functional theory (DFT) at the B3LYP/6-31+G(d) level using the Gaussian 09 software (Gaussian, Inc., Wallingford, CT 06492, United States) and the calculated VCD spectrum of the (6*R*, 7*R*) configuration showed good agreement with the experimental spectrum of **1**, with a confidence level of 87% (Figure 4).

Bazzanane-type sesquiterpenes have been mainly found in liverworts, including the *Bazzania* genus. After the first reported isolation of bazzanene from *Bazzania pompeana* (Lac.) Mitt. in 1969 [22], only a few bazzanane-type sesquiterpenes have been reported, indicating that they are very rare in nature [23,24]. The most similar compound to **1** was isolated from the New Zealand liverwort *Frullania falciloba* [25]. However, previously reported bazzanane-type sesquiterpenes are neither chlorinated nor possess an amide group in the molecule, as in **1**. Furthermore, **1** is the first bazzanane-type sesquiterpenoid to be isolated from a strain of the genus *Acremonium* and the first reported chlorinated bazzanane-type sesquiterpene with an amide group in its structure.

**Figure 5:**
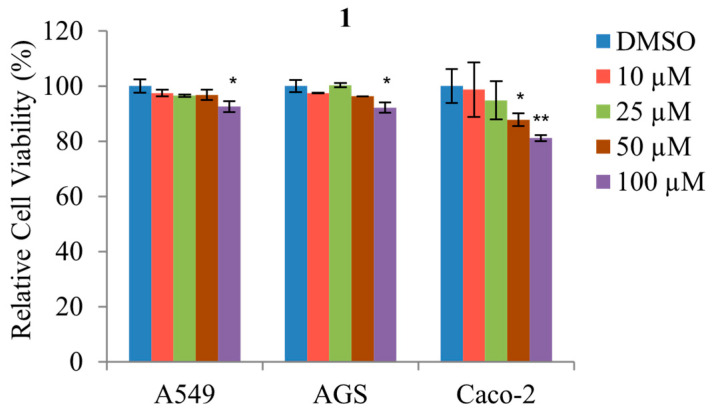
The effects of various concentrations of **1** upon the cell viability of A549, AGS, and Caco-2. Cell viability was measured using MTT assay. The data represent the mean ± standard deviation, *n* = 3. * *p* < 0.05; ** *p* < 0.01.

A methyl thiazolyl tetrazolium (MTT) cytotoxicity bioassay was used to evaluate the effect of **1** treatment in various concentrations (10, 25, 50, and 100 μM) on the viability of AGS (gastric cancer), A549 (lung cancer), and Caco-2 (colorectal cancer) cells. The cell viability of A549 was unaffected during the treatment with 10–50 μM of **1** for 48 h; however, the viability decreased marginally during the treatment with 100 μM of **1**, as shown in Figure 5. Similarly, the viability of AGS and Caco-2 did not decrease with treatment of 10–25 μM of **1** but decreased significantly at concentrations of 50–100 μM. Thus, these observations demonstrate that treatment with **1** is relatively non-toxic toward A549, AGS, and Caco-2 cells at concentrations less than 25 μM.

**Figure 6:**
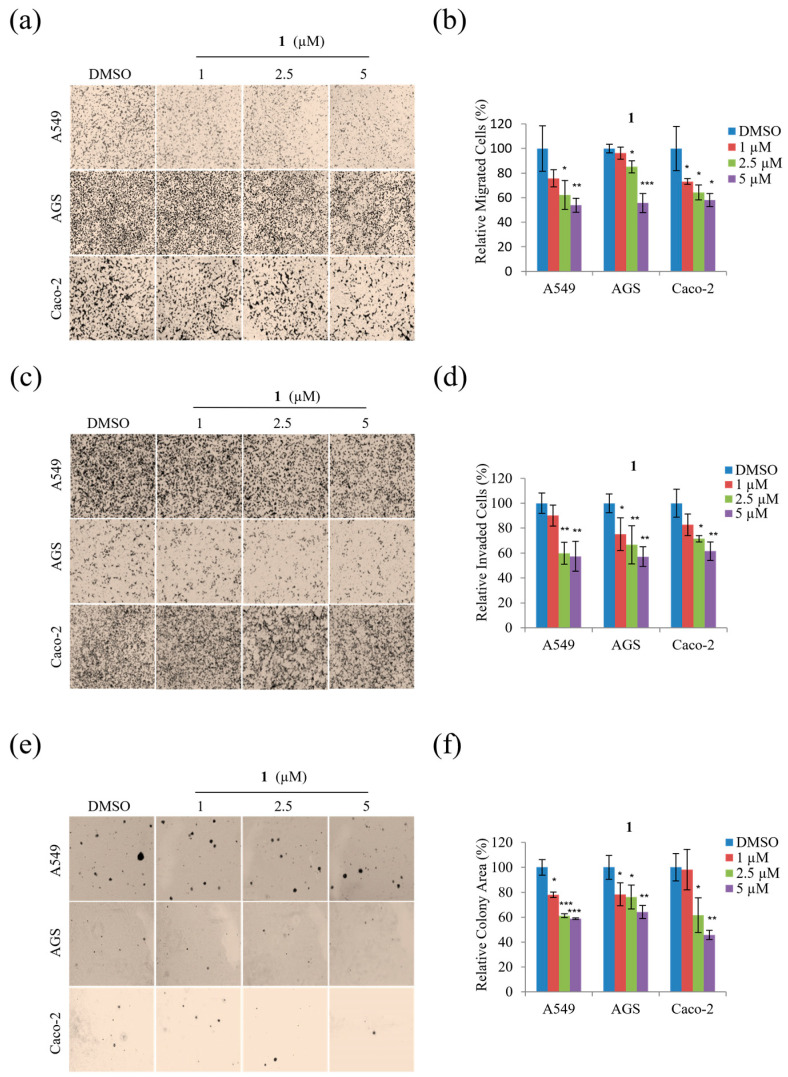
The effects of **1** on the motility and tumorigenicity of A549, AGS, and Caco-2 cells: (**a**) representative images of each insert in the migration assay; (**b**) relative percentage of migrated cells; (**c**) representative images of each insert in the invasion assay; (**d**) relative percentage of invaded cells; (**e**) representative images of colonies formed in the soft agar assay; (**f**) relative surface area of colonies. Data represent the mean ± standard deviation, *n* = 3. * *p* < 0.05; ** *p* < 0.01; *** *p* < 0.001.

Next, migration and invasion assays were performed using non-toxic concentrations (1, 2.5, and 5 μM) to determine whether **1** inhibits the motility of A549, AGS, and Caco-2 cells. As shown in Figure 6, **1** displayed a dose-dependent inhibitory effect on the migration of all three cell types at concentrations from 1 to 5 µM (Figure 6a,b) alongside dose-dependent inhibition of invasion by each cell type by up to ~45% at 5 µM concentrations (Figure 6c,d).

In addition, the potential anti-tumorigenic activity of **1** was evaluated by examining the soft agar colony formation of A549, AGS, and Caco-2 cells exposed to non-toxic concentrations (1, 2.5, and 5 µM). As shown in Figure 6e,f, compound **1** dose-dependently decreased the colony formation of A549, AGS, and Caco-2 cells. Moreover, Figure 6f reveals that treatment with 5 µM of **1** significantly decreased the tumorigenicity of A549, AGS, and Caco-2 cells. Overall, these results demonstrate that treatment with 1, 2.5, and 5 µM concentrations of **1** significantly suppressed the motility and tumorigenicity of A549, AGS, and Caco-2 cells.

**Figure 7:**
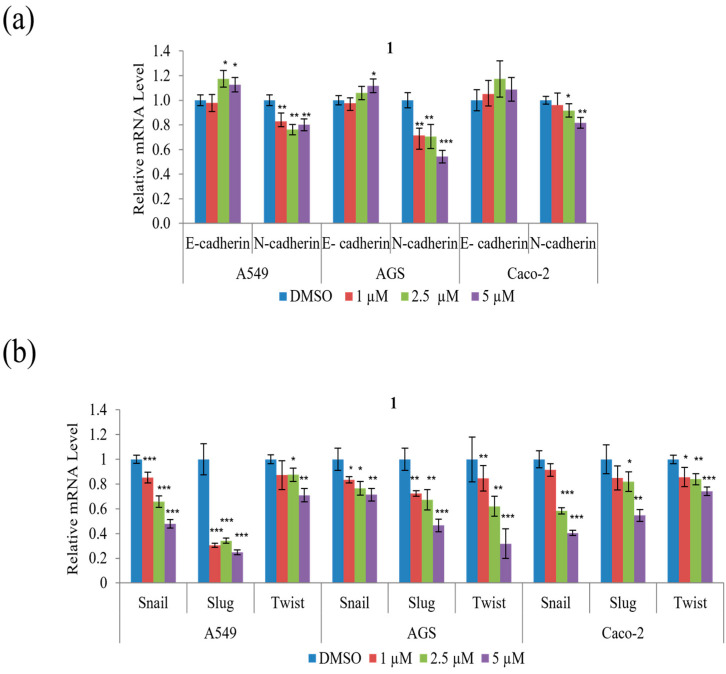
The effects of **1** on the expression of EMT effectors and transcription factors in A549, AGS, and Caco-2 cells: (**a**) relative mRNA expression of the EMT effectors N-cadherin and E-cadherin; (**b**) relative mRNA expression of the EMT transcription factors Snail, Slug, and Twist. The mRNA levels were normalized against that of glyceraldehyde 3-phosphate dehydrogenase (GAPDH). Data represent the mean ± standard deviation, *n* = 3. * *p* < 0.05; ** *p* < 0.01; *** *p* < 0.001.

To determine whether the suppression of A549, AGS, and Caco-2 cell motility and tumorigenicity in the presence of **1** involves the epithelial–mesenchymal transition (EMT), the expression of EMT effectors and transcription factors were examined. As shown in Figure 7a, **1** decreased mRNA expression of the mesenchymal marker N-cadherin but increased that of the epithelial marker E-cadherin in all three cell types. Further, **1** significantly decreased the mRNA expression of the EMT transcription factors, Snail, Slug, and Twist, in all three cell types, as shown in Figure 7b. In summary, these results indicate that **1** modulates the expression of the EMT effector N-cadherin by downregulating the transcription factors Snail, Slug, and Twist.

**Figure 8:**
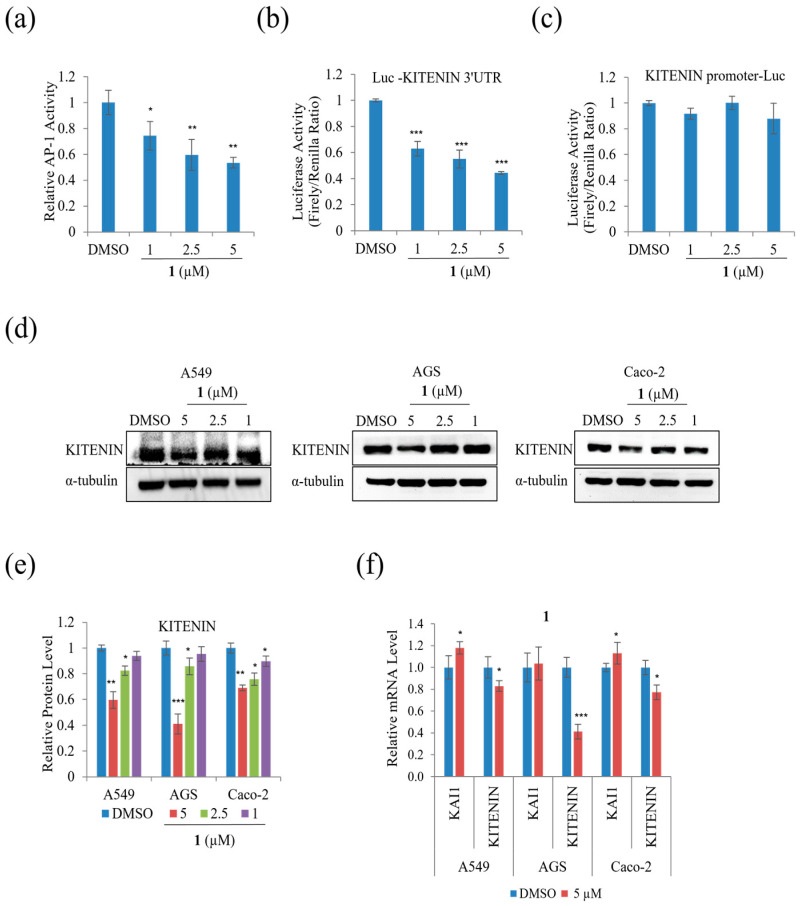
The effects of **1** on the AP-1 activity, KITENIN, and KAI1 in A549, AGS, and Caco-2 cells: (**a**) relative AP-1 activity in HEK293T cells; (**b**) 3-UTR luciferase activity in HEK293T cells; (**c**) KITENIN promoter luciferase activity in HEK293T; (**d**) Western blot analysis of KITENIN in A549, AGS, and Caco-2 cells; (**e**) relative protein levels in A549, AGS, and Caco-2 cells; (**f**) relative mRNA expression of the KITENIN and KAI1 in A549, AGS, and Caco-2 cells. Data represent the mean ± standard deviation, *n* = 3. * *p* < 0.05; ** *p* < 0.01; *** *p* < 0.001.

To examine whether the suppression of A549, AGS, and Caco-2 cell motility and tumorigenicity in the presence of **1** involves KITENIN and AP-1, the protein and mRNA expression levels of KITENIN and AP-1, including their activities, were examined. Epidermal growth factor increases KITENIN-mediated AP-1 activity, and there is an inverse relationship between KAI and KITENIN [26]. As shown in Figure 8a, at a concentration of 5 μM, **1** suppressed AP-1 activity. Figure 8b,c show that **1** suppressed the activity of the KITENIN 3′-UTR, while the KITENIN promoter did not show significant change. Figure 8d,e indicates that the protein level of KITENIN decreased in A549, AGS, and Caco-2 during treatment. The mRNA expression level of KITENIN was also suppressed in A549, AGS, and Caco-2, whereas the mRNA level of KAI1 was increased by **1** (Figure 8f). As a result, **1** decreased cell motility by downregulating the expression level of KITENIN while upregulating that of KAI1.

**Figure 9:**
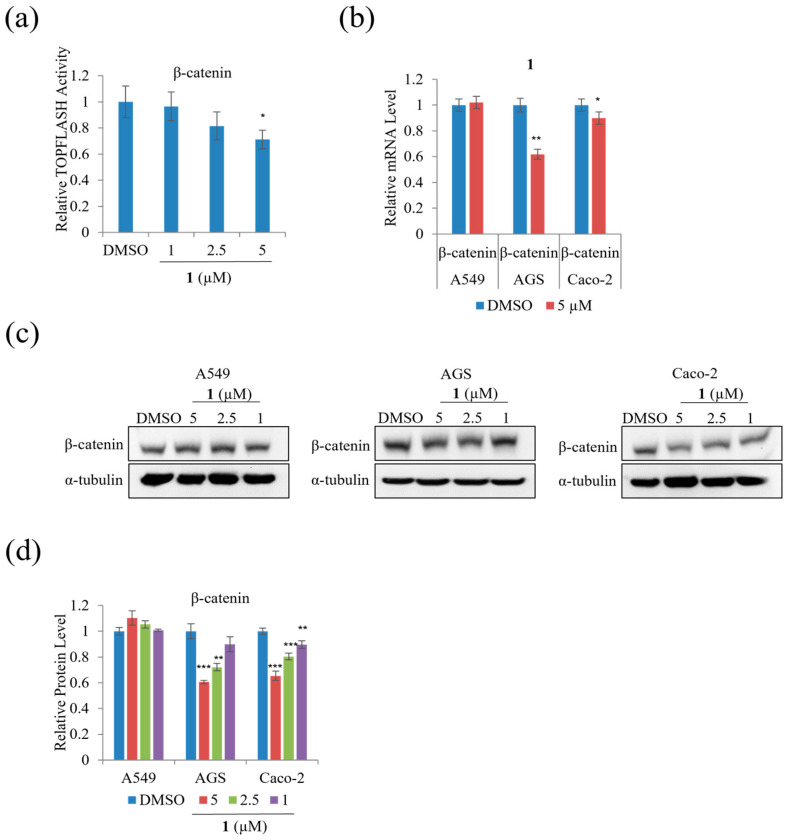
The effects of **1** on the β-catenin-mediated TOPFLASH activity: (**a**) the β-catenin-mediated TOPFLASH activity in HEK293T cells; (**b**) relative β-catenin in A549, AGS, and Caco-2 cells; (**c**) Western blot analysis of β-catenin in A549, AGS, and Caco-2 cells; (**d**) relative protein levels of β-catenin. Data represent the mean ± standard deviation, *n* = 3. * *p* < 0.05; ** *p* < 0.01; *** *p* < 0.001.

We performed TOPFLASH reporter assays to assess whether treatment with **1** modulates β-catenin-mediated and/or KITENIN-mediated signaling activity. Treatment with 5 µM of **1** significantly decreased β-catenin-mediated TOPFLASH activity on HEK293T by 30% (Figure 9a). In addition, Figure 9b–d indicates that treatment with **1** decreased mRNA expression and the protein level of β-catenin on AGS and Caco-2 cells; however, treatment with **1** did not significantly affect the mRNA expression and protein level of β-catenin in A549 cells.

**Figure 10:**
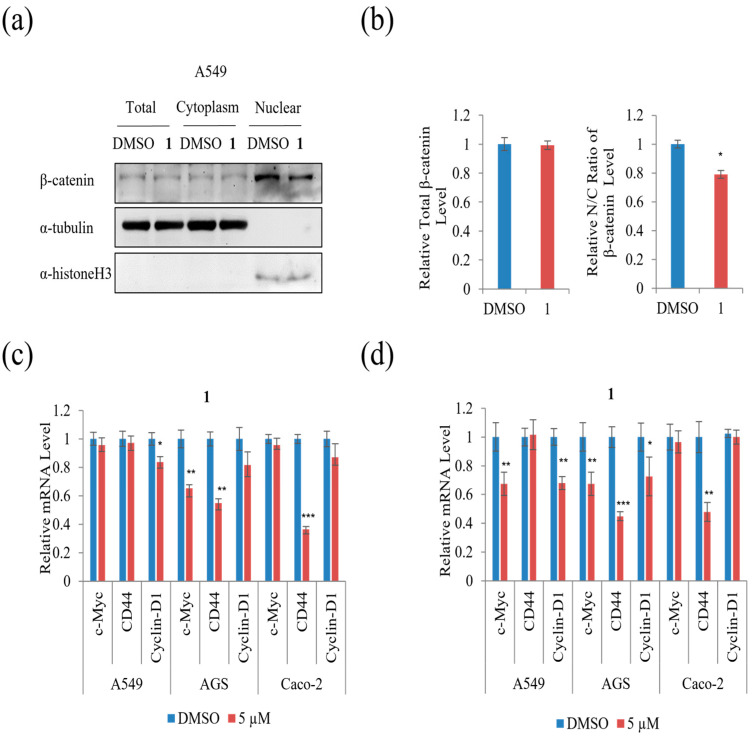
The effects of **1** β-catenin-mediated TOPFLASH activity by suppressing nuclear import: (**a**) relative protein level of total cytoplasmic and nuclear β-catenin in A549 cells. α-Histone 3 served as loading control; (**b**) Western blot analysis of total cytoplasmic and nuclear β-catenin in A549 cells; (**c**) relative of the mRNA level of c-Myc, CD44, and cyclin-D1 in A549, AGS, and Caco-2 cells for 24 h and (**d**) relative of the mRNA level of c-Myc, CD44, and cyclin-D1 in A549, AGS, and Caco-2 cells for 48 h. Data represent the mean ± standard deviation, *n* = 3. * *p* < 0.05; ** *p* < 0.01; *** *p* < 0.001.

The protein level of total, cytoplasmic, and nuclear β-catenin was examined to test whether **1** affected the nuclear/cytoplasmic distribution of β-catenin in A549 cells. As shown in Figure 10a,b, treatment with 5 µM of **1** did not affect the level of total β-catenin, whereas it decreased the β-catenin nuclear to cytoplasmic ratio remarkably in A549 cells compared to DMSO. To further test the effect of **1** on the levels of downstream target genes of β-catenin, qRT-PCR analysis was performed. As shown in Figure 10c, treatment with 5 µM of **1** suppressed the mRNA expression of cyclin-D1 in A549 and CD44 in Caco-2, while treatment with 5 µM of **1** suppressed the mRNA expression of c-Myc and CD44 in AGS cells for 24 h. Moreover, the β-catenin downstream target genes, including c-Myc, CD44, and cyclin-D1, were suppressed in AGS cells during treatment with 5 µM of **1** for 48 h (Figure 10d). Treatment with 5 µM of **1** suppressed the mRNA expression of cyclin-D1 and CD44 in A549, while 5 µM of **1** suppressed the mRNA expression of CD44 in Caco-2 cells for 48 h (Figure 10d). These results show that **1** decreased β-catenin-mediated TOPFLASH activity and its downstream targets in three cancer cells.

**Figure 11:**
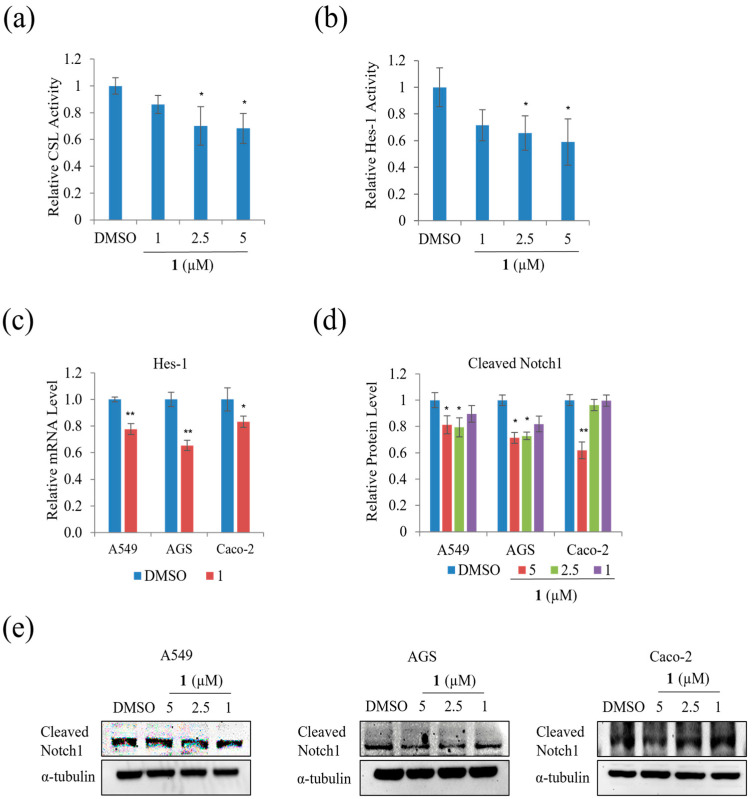
The effects of **1** on the Notch signal pathway in A549, AGS, and Caco-2 cells: (**a**) relative CSL activity in HEK293T cells; (**b**) relative Hes-1 activity in HEK293T cells; (**c**) relative mRNA expression of the Hes-1 in A549, AGS, and Caco-2 cells; (**d**) Western blot analysis of cleaved Notch1 in A549, AGS, and Caco-2 cells and (**e**) relative protein level in Hes-1 in A549, AGS, and Caco-2 cells. Data represent the mean ± standard deviation, *n* = 3. * *p* < 0.05; ** *p* < 0.01.

We examined the effect of **1** on the Notch signal pathway in A549, AGS, and Caco-2 cells and found that treatment with 5 µM of **1** significantly decreased the relative CSL activity in HEK293T cells by approximately 20% (Figure 11a). In addition, the relative Hes-1 activity in HEK293T and mRNA expression of Hes-1 in AGS, A549, and Caco-2 cells decreased during treatment with 5 µM, as shown in Figure 11b,c. The level of expression of HES correlated with NICD and CSL. Treatment with **1** decreased the level of Cleaved Notch1 in A549, AGS, and Caco-2 cells (Figure 11d,e). This result indicates that **1** slightly suppressed the Notch signal pathway in A549, AGS, and Caco-2 cells.

**Figure 12:**
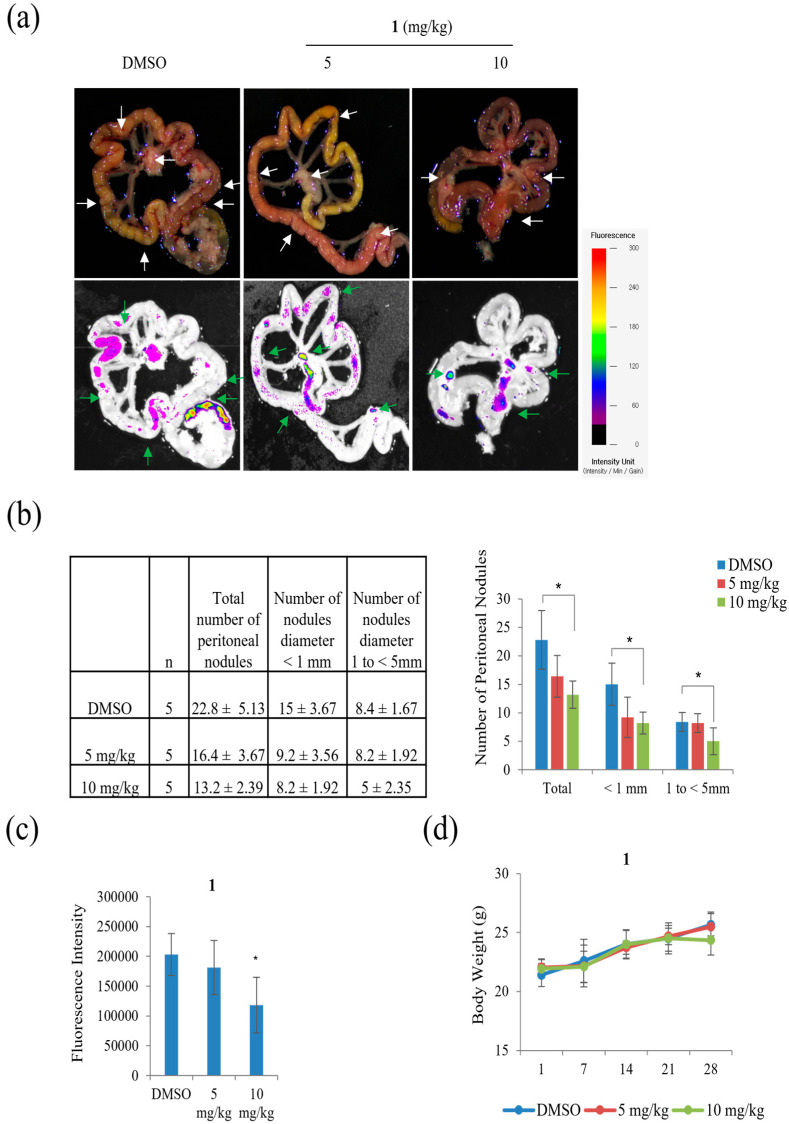
The effects of **1** on peritoneal metastasis of gastric cancer. AGS cells (1 × 10^7^ per mouse) were injected into the abdominal cavities of mice: (**a**) fluorescence representative images obtained from an organ bioimaging instrument (FOBI) system and appearance of peritoneal tumors established by intraperitoneal inoculation of AGS human gastric-cancer cells; (**b**) the number of peritoneal nodules in each group; (**c**) fluorescence area of images obtained from a fluorescence-labeled organ bioimaging instrument (FOBI) system; (**d**) body weight of control and **1** treated mice. Data represent the mean ± standard deviation, *n* = 5. * *p* < 0.05.

Peritoneal carcinomatosis occurs when gastric cancer metastasizes to the peritoneal cavity [27]. Peritoneal carcinomatosis is a hallmark of advanced peritoneal tumor progression, and peritoneal recurrence from gastric cancer occurs due to resistance to chemotherapy. On day 28 after inoculation of AGS-iRFP, treatment of 10 mg/kg of **1** significantly reduced the three different categories of the number of tumor nodules in the mesentery compared to the control (Figure 12). As shown in Figure 12b, treatment with 10 mg/kg of **1** reduced the number of nodules with diameters >1 compared to other groups (control group, 8.4 ± 1.67 and 5 mg/kg of **1** group, 8.2 ± 1.92 versus 10 mg/kg of **1**, 5 ± 2.35, *p* < 0.05). The number of nodules with diameters ranging from 1 to < 5 mm in the group with 10 mg/kg of **1** was also lower than those in the other groups (control group, 8.4 ± 1.67 and 5 mg/kg of **1** group, 8.2 ± 1.92 versus 10 mg/kg of **1**, 5 ± 2.35, *p* < 0.05). As a result, the quantitative data showed that the total number of metastatic nodules was significantly reduced in the mice treated with 10 mg/kg of **1** compared to those in the control group (Figure 12c).

## 3. Materials and Methods

### 3.1. General Experimental Procedures

Optical rotation was acquired using a Kruss Optronic P-8000 polarimeter with a 5-cm cell. The UV spectrum was recorded in methanol (MeOH) on a Scinco UVS2100, and the VCD spectra were measured using a BioTools dualPEM ChiralIR spectrophotometer. The IR spectrum was collected on a Varian Scimitar Series. The ^1^H and 2D NMR spectra were recorded at 400 and 500 MHz in CD_3_OD, containing Me_4_Si as the internal standard on Varian Inova spectrometers. The ^13^C NMR spectra were acquired at 75 MHz on a Varian Inova spectrometer. The high-resolution mass spectrum was obtained on a JMS-700 (JEOL) mass spectrometer, and the low-resolution LC-MS data were measured using the Agilent Technologies 1260 quadrupole and Waters Micromass ZQ LC/MS system with a reversed-phase column (Phenomenex Luna C_18_ (2), 100 Å, 50 mm × 4.6 mm, 5 µm), at a flow rate of 1.0 mL/min, at the National Research Facilities and Equipment Center (NanoBioEnergy Materials Center) at Ewha Womans University. Medium-pressure liquid chromatography (MPLC) was performed on a Biotage Isolera One system (SE-751 03 Uppsala, Sweden), using Biotage SNAP KP-Sil, with a step gradient solvent of dichloromethane (DCM) and methanol (MeOH). The fractions were purified by reversed-phase high-performance liquid chromatography (HPLC) (Phenomenex Luna C_18_ (2), 100 Å, 250 nm × 10 mm, 5 μm).

### 3.2. Collection and Phylogenetic Analysis of the Strain CNQ-049

The marine-derived *Acremonium* sp. CNQ-049 was isolated from marine sediment collected off the coast of Southern California. The strain CNQ-049 was identified as *Acremonium* sp. with 99.4% similarity to that of *Acremonium fusidiodes*, based on 18S rRNA gene sequence analysis (GenBank accession number KP131520.1).

### 3.3. Fermentation, Extraction, and Isolation

Strain CNQ-049 was cultured in 80 × 2.5 L Ultra Yield flasks, each containing 1 L of the medium (10 g/L soluble starch, 2 g/L yeast, 4 g/L peptone, and 34.75 g/L sea salt dissolved in distilled water), and shaken at 120 rpm and 27 °C. After seven days of cultivation, the broth was extracted with ethyl acetate (EtOAc) (80 L overall), and the soluble fraction was dried in vacuo to afford 5 g of the crude extract. This was then separated on a MPLC silica-gel column (Biotage^®^ SNAP Cartridge, KP-SIL), with step gradients of MeOH/DCM (0 to 100%), to obtain 10 fractions, which were labeled as Q049-1−Q049-10. Fraction Q049-6 (762 mg) was separated into six subfractions, labeled Q049-6-A−Q049-6-F, by C_18_ reversed-phase column chromatography with 37% aqueous acetonitrile. Subfraction Q049-6-B (221 mg) was further purified by reversed-phase HPLC (Phenomenex Luna C_18_ (2), 250 × 100 mm, 2.0 mL/min, 5 μm, 100 Å, UV = 210 nm) with 38% CH_3_CN to obtain 10.7 mg of marinobazzanan (**1**).

Marinobazzanan (**1**): pale-yellow amorphous powder; ${\left[\alpha \right]}_{D}^{21}\;$−29 (c 0.0625, MeOH); UV (MeOH) λ_max_ (log *ε*) 205 (3.73) nm; IR (KBr) ν_max_ 3338, 2964, 2935, 2360, 1669, 1373, 1082 cm^−1^; ^1^H NMR and 2D NMR (400 MHz and 500 MHz, CD_3_OD) see Table 1; HR-ESI-MS *m/z* 284.1415 [M + H]^+^ (calcd for C_15_H_22_^35^ClNO_2_, 284.1417).

### 3.4. MTPA Esterification of Marinobazzanan

To obtain the (*S*)- and (*R*)-MTPA esters, 2.0 mg of **1** was completely dried under high vacuum for 12 h and dissolved in dried pyridine (0.6 mL). Catalytic amounts of crystalline 4-dimethylaminopyridine (DMAP) were added and, respectively, treated with (*R*)-and (*S*)-*α*-methoxy trifluoromethyl-phenylacetic acid (MTPA) chloride (6 μL). The mixtures were stirred for 12 h at 50 °C and purified by reversed-phase HPLC (Phenomenex Luna C_18_ (2), 250 × 100 mm, 2.0 mL/min, 5 μm, 100 Å), with 90% aqueous acetonitrile, to afford 0.8 mg and 0.7 mg of (*S*)- and (*R*)-MTPA esters of **1**, respectively.

(*S*)-MTPA ester of **1**: ^1^H NMR (500 MHz, in CDCl_3_) *δ*_H_ 0.88 (3H, s, H-14), 1.23 (3H, s, H-13), 1.41–1.61 (2H, overlapped, H-1), 1.86 (1H, dd, *J* = 13.0, 10.0 Hz, H-11a), 2.02 (1H, dd, *J* = 13.0, 7.5 Hz, H-11b), 1.99 (1H, m, H-5a), 2.12 (1H, m, H-5b), 2.21 (1H, m, H-2a), 2.29 (1H, m, H-2b), 4.46 (1H, dt, *J* = 10.0, 2.7 Hz, H-9), 5.22 (1H, d, *J* = 2.7 Hz, H-15a), 5.24 (1H, dt, *J* = 10.0, 7.5 Hz, H-10), 5.52 (1H, d, *J* = 2.7 Hz, H-15b), 6.55 (1H, br. s, H-4); LR-ESI-MS *m/z* 457.3 [M − CONH_2_ + H]^+^.

(*R*)-MTPA ester of **1**: ^1^H NMR (500 MHz, in CDCl_3_) *δ*_H_ 0.84 (3H, s, H-14), 1.22 (3H, s, H-13), 1.38−1.60 (2H, overlapped, H-1), 1.67 (1H, dd, *J* = 13.0, 10.0 Hz, H-11a), 2.00 (1H, dd, *J* = 13.0, 7.5 Hz, H-11b), 1.95 (1H, m, H-5a), 2.07 (1H, m, H-5b), 2.18 (1H, m, H-2a), 2.26 (1H, m, H-2b), 4.50 (1H, dt, *J* = 10.0, 2.7 Hz, H-9), 5.20 (1H, dt, *J* = 10.0, 7.5 Hz, H-10), 5.22 (1H, d, *J* = 2.7 Hz, H-15a), 5.54 (1H, d, *J* = 2.7 Hz, H-15b), 6.52 (1H, br. s, H-4); LR-ESI-MS *m/z* 457.3 [M − CONH_2_ + H]^+^.

### 3.5. VCD Analysis and Calculations

The conformational assignments for the C-6 and C-7 positions of **1** were performed using the Macromodel software (Version 9.9, Schrodinger LLC.) with “Mixed torsional/Low-mode sampling” in the GAFF force field. The experiments were conducted in the gas phase with the 50 kJ/mol energy window limit and a maximum of 10,000 steps to thoroughly examine all low-energy conformers. The Polak–Ribière conjugate gradient method was utilized for the minimization processes with 10,000 maximum iterations and a 0.001 kJ (mol Å)^−1^ convergence threshold on the root mean square gradient. Conformers within 10 kJ/mol of each global minimum for the 6*R*,7*R*, and 6*S*,7*S* forms of **1** were used for calculating the gauge-independent atomic orbital shielding constant, without geometric optimization, by employing the TmoleX Version 4.2.1 software (COSMOlogic GmbH & Co. KG) at the B3LYP/6-31 + G(d) level in the gas phase. A sample of **1** (5.0 mg) was dissolved in CDCl_3_ (150 μL) and placed in a BaF2 cell with a path length of 100 μm, and data were acquired on a BioTools dualPEM ChiralIR spectrophotometer. The spectra were collected in 12 blocks, and each block was acquired for 3120 scans.

### 3.6. Cell Culture

The human cancer-cell lines A549 (lung cancer), AGS (gastric cancer), and Caco-2 (colorectal cancer) were cultured in Roswell Park Memorial Institute (RPMI) 1640 Medium or Dulbecco’s Modified Eagle Medium (DMEM) (Gen Depot, Barker, TX, USA), supplemented with 10% FBS and 1% penicillin–streptomycin solution in a humidified atmosphere containing 5% CO_2_ at 37 °C [28].

### 3.7. Methyl Thiazolyl Tetrazolium (MTT) Assay

Marinobazzanan (**1**) was dissolved in dimethyl sulfoxide (DMSO, Sigma-Aldrich, St.Louis, MO, USA) and diluted to four concentrations (10, 25, 50, and 10 µM). The cells were seeded on 96-well plates (3 × 10^3^ cells/well) for 12–16 h and then treated with 10, 25, 50, and 10 µM of compound **1** for 48 h [29]. After 4 h of incubation with MTT in 5% CO_2_ at 37 °C, the cells were lysed with 150 µL of DMSO (Sigma-Aldrich), and the absorbance was measured at 570 nm using a spectrophotometer (Bio Tek Instruments, Winooski, VT, USA).

### 3.8. Invasion Assay

The invasion of cancer cells was measured using Transwell chambers (Corning, New York, NY, USA) [30] containing polycarbonate membranes with 8 µm pores coated with 1% gelatin. The AGS (3 × 10^5^), A549 (3 × 10^5^), and Caco-2 (2.5 × 10^5^) cells were seeded in a culture medium containing 0.2% bovine serum albumin (BSA) and incubated with 1, 2.5, and 5 µM of compound **1** or DMSO control for 24 h. The lower chamber was filled with 600 μL DMEM/RPMI containing 0.2% BSA and 10 μg/mL fibronectin (EMD Millipore Corp., Billerica, MA, USA) as a chemoattractant. After 24 h of incubation, the invading cells were fixed using a Diff-Quik kit (Sysmex, Kobe, Japan). The number of cells was quantified using a Nikon Eclipse 400 fluorescence microscope (Nikon Instech, Co., Ltd., Kawasaki, Japan) and i-Solution FL Auto Software (IMT i-Solution Inc., Vancouver, QC, Canada; five fields/chamber).

### 3.9. Migration Assay

Migration assays were performed in non-coated Transwell chambers [30]. The cells were seeded at a density of 2.5–3 × 10^5^ cells/well in RPMI 1640/DMEM containing 0.2% BSA in the upper compartment of the chamber. The lower chamber was filled with 600 μL RPMI 1640/DMEM containing 0.2% BSA and fibronectin as a chemoattractant. The cells were cultured either in the absence or presence of compound **1** (1, 2.5, and 5 µM) for 24 h and were fixed using a Diff-Quick kit. The cells in the upper chamber were counted using a Nikon Eclipse 400 fluorescence microscope (Nikon Instech, Co., Ltd.) and i-Solution FL Auto Software (IMT i-Solution Inc.; five fields/chamber)

### 3.10. Soft Agar Colony Formation Assay

The cancer cells were suspended at a density of 2.5–3 × 10^3^ cells/well in 1.0 mL of soft agar (0.35% soft-agar solution diluted 2-fold with 2 × DMEM/RPMI) and planted onto 1 mL of soft agar (0.5% agarose solution diluted 2-fold with 2 × DMEM/RPMI) in a 12-well plate and cultured for three weeks [31]. The cells were fed twice per week with cell-culture media, compound **1** (1, 2.5, and 5 µM), or DMSO. The surface areas of the colonies in the five fields per well were estimated using a Nikon Eclipse 400 fluorescence microscope (Nikon Instech, Co., Ltd.) and i-Solution FL Auto Software (IMT i-Solution Inc.; five fields/chamber). Three replications were performed.

### 3.11. Quantitative Real-Time PCR

The total RNA was isolated from A549, AGS, and Caco-2 cells using RNAiso Plus (Takara, Otsu, Japan) according to the manufacturer’s instructions. Moloney murine leukemia virus reverse transcriptase (Invitrogen, Carlsbad, CA, USA) was used to convert 1 μg of RNA into cDNA. The dye SYBR Green (Enzynomics, Seoul, Republic of Korea) was used to analyze relative gene expression. Further, the qRT-PCR reaction and analysis were performed using CFX (Bio-Rad, Hercules, CA, USA).

### 3.12. Western Blotting

The A549, AGS, and Caco-2 cells were treated with **1** for 24 h, and washed twice with ice-cold phosphate-buffered saline (PBS). Lysis buffer was used for extraction. In some experiments, cytoplasmic and nuclear extracts were separated with the NE-PER nuclear and cytoplasmic extraction kit (Pierce Biotechnology, USA), and the extracted protein was separated using SDS-PAGE. The density of the bands was measured using the Multi Gauge 3.0 (Fujifilm, Tokyo, Japan) software, and the bands’ relative density was calculated based on the density of the control bands during loading in each sample.

### 3.13. Reporter Assay

HEK293T was seeded in 24-well plates. Following attachment, the cells were transfected with TOPFLASH, AP1, KITENIN, CSL, HES, NF-κb, KITENIN 3′-untranslated region (3′-UTR) reporters with renilla luciferase reporter plasmid (pRL-TK). After 12 h transfection of these reporters, the cells were treated with 1 µM, 2.5 µM, and 5 µM of compound **1**. The Luciferase activity was calculated using the Dual-Luciferase Reporter Assay (Promega, Wisconsin, USA) and normalized to Renilla luciferase.

### 3.14. Lentiviral Transduction

The human gastric-cancer AGS cells were cultured in Roswell Park Memorial Institute (RPMI) 1640 Medium (Gen Depot, Barker, TX, USA), supplemented with puromycin solution. After 12 h, the lentiviral vector was transfected into the human gastric-cancer cell line by polybrene, and the returned cells were incubated. After 72 h, the single cells were then plated in individual wells of a 96-well plate and incubated for 7–10 days.

### 3.15. Animal Studies

The AGS-iRFP cell suspension (1 × 10^7^cells in 0.1 mL PBS per mouse) was implanted into the abdominal cavity of male BALB/c nude mice (five weeks old), obtained from Central Lab. Animal Inc. (Seoul, Korea). All in-vivo experiments were performed according to the Guiding Principles for the Care and Use of Animals (DHEW publication, NIH 80-23) and were approved by the Sunchon National University Research Institutional Animal Care and Use Committee. The mice were randomly assigned to one of three groups: control, 5 mg/kg of **1**, and 10 mg/kg of **1** by IP treatment. All animals were examined by measuring weight change. The treatment was initiated a week after the AGS-iRFP cells were injected. On day 28 after tumor inoculation, all the mice were sacrificed. The fluorescence area of the images alongside the representative images was then obtained through a fluorescence-labeled organism bioimaging instrument system.

## 4. Conclusions

In conclusion, this study described a new bazzanane-type sesquiterpenoid, marinobazzanan (**1**), from the genus *Acremonium*. This is the first *Acremonium*-derived bazzanane-type sesquiterpenoid isolated with chemical-structure modifications, such as chlorination and amination. Marinobazzanan (**1**) was shown to inhibit cancer-cell migration and invasion at non-toxic concentrations of 1, 2.5, and 5 µM and downregulate the transcription factors Snail, Slug, and Twist. In addition, marinobazzanan (**1**) decreased cell motility by downregulating the expression level of KITENIN while upregulating that of KAI1. Furthermore, the new compound modulated the expression of β-catenin by downregulating downstream target genes. Marinobazzanan (**1**) was also shown to have a reduced number of metastatic nodules in an intraperitoneal xenograft mouse model. Together, these findings suggest that **1** exhibits potent anticancer activity against cancer cells in vitro and anti-cancer activity for the peritoneal carcinomatosis model in vivo.

## Figures and Tables

**Table 1:** NMR Data for Marinobazzanan (**1**) (CD_3_OD) ^a^.

Position	*δ*_C_, Type	*δ*_H_, Mult ^b^ (*J* in Hz)	COSY	HMBC
1	27.4, CH_2_	1.65, m	2	C-2, 6, 7, 14
		1.54, m		
2	21.8, CH_2_	2.39, m	1	C-1, 3, 4, 6
		2.21, m		
3	131.3, qC			
4	134.0, CH	6.66, d (4.8)	5	C-5, 2, 6, 12
5	33.0, CH_2_	2.21, m	4	C-1, 3, 4, 6, 14
		2.05, br. D (18.0)		
6	36.8, qC			
7	48.5, qC			
8	153.7, qC			
9	69.2, CH	4.29, dt (10.0, 2.7)	10	C-8, 10, 15
10	75.6, CH	3.86, td (10.0, 7.5)	9, 11	C-9, 11
11	40.5, CH_2_	1.87, dd (13.0, 10.0)	10	C-6, 7, 8, 9, 10, 13
		1.79, dd (13.0, 7.5)		
12	172.2, qC			
13	24.5, CH_3_	1.20, s		C-6, 7, 8, 11
14	17.1, CH_3_	0.92, s		C-1, 5, 6, 7
15	111.9, CH_2_	5.44, d (2.7)		C-7, 8, 9
		5.21, d (2.7)		

^a^ 400 MHz for ^1^H NMR and 75 MHz for ^13^C NMR. ^b^ Numbers of attached protons were determined by analysis of 2D spectra.

## Data Availability

Not applicable.

## Supplementary Materials

The following supporting information can be downloaded at: https://www.mdpi.com/article/10.3390/md21030153/s1, Figure S1: ^1^H NMR spectrum (400 MHz) of marinobazzanan (**1**) in CD_3_OD; Figure S2: ^13^C NMR spectrum (75 MHz) of marinobazzanan (**1**) in CD_3_OD; Figure S3: COSY NMR spectrum (500 MHz) of marinobazzanan (**1**) in CD_3_OD; Figure S4: HSQC NMR spectrum (500 MHz) of marinobazzanan (**1**) in CD_3_OD; Figure S5: HMBC NMR spectrum (500 MHz) of marinobazzanan (**1**) in CD_3_OD; Figure S6: NOESY NMR spectrum (500 MHz) of marinobazzanan (**1**) in CD_3_OD; Figure S7: ^1^H NMR spectrum (500 MHz) of *S*-MTPA ester (**1a**) for marinobazzanan (**1**) in CDCl_3_; Figure S8: ^1^H NMR spectrum (500 MHz) of *R*-MTPA ester (**1b**) for marinobazzanan (**1**) in CDCl_3_; Figure S9: The effect of various fractions of marinobazzanan (**1**) upon the cell viability of Caco2; Figure S10: LRMS spectrum of marinobazzanan (**1**).

## References

[1] Sung H., Ferlay J., Siegel R.L., Laversanne M., Soerjomataram I., Jemal A., Bray F. (2021). Global Cancer Statistics 2020: GLOBOCAN Estimates of Incidence and Mortality Worldwide for 36 Cancers in 185 Countries. CA Cancer J. Clin..

[2] Le T.C., Pulat S., Lee J., Kim G.J., Kim H., Lee E.-Y., Hilman P.F., Choi H., Yang I., Oh D.-C. (2022). Marine Depsipeptide Nobilamide I Inhibits Cancer Cell Motility and Tumorigenicity via Suppressing Epithelial-Mesenchymal Transition and MMP2/9 Expression. ACS Omega.

[3] Ribatti D., Tamma R., Annese T. (2020). Epithelial-Mesenchymal Transition in Cancer: A Historical Overview. Transl. Oncol..

[4] Shang S., Hua F., Hu Z.-W. (2017). The regulation of β-catenin activity and function in cancer: Therapeutic opportunities. Oncotarget.

[5] Joo Y.-E., Ryu H.-S., Park Y.-L., Park S.-J., Lee J.-H., Cho S.-B., Lee W.-S., Chung I.-J., Kim K.-K., Lee K.-H. (2010). KITENIN is associated with tumor progression in human gastric cancer. Anticancer. Res..

[6] Xiao W., Zheng S., Xie X., Li X., Zhang L., Yang A., Wang J., Tang H., Xie X. (2020). SOX2 Promotes Brain Metastasis of Breast Cancer by Upregulating the Expression of FSCN1 and HBEGF. Mol. Ther. Oncolytics.

[7] Amend A., Burgaud G., Cunliffe M., Edgcomb V.P., Ettinger C.L., Gutiérrez M.H., Heitman J., Hom E.F.Y., Ianiri G., Jones A.C. (2019). Fungi in the Marine Environment: Open Questions and Unsolved Problems. mBio.

[8] Tasdemir D. (2017). Marine fungi in the spotlight: Opportunities and challenges for marine fungal natural product discovery and biotechnology. Fungal Biol. Biotechnol..

[9] Pang K.L., Overy D.P., Jones E.B.G., da Luz Calado M., Burgaud G., Walker A.K., Johnson J.A., Kerr R.G., Cha H.-J., Bills G.F. (2016). ‘Marine fungi’ and ‘marine-derived fungi’ in natural product chemistry research: Toward a new consensual definition. Fungal Biol. Rev.

[10] Imhoff J.F. (2016). Natural Products from Marine Fungi—Still an Underrepresented Resource. Mar. Drugs.

[11] Bhadury P., Mohammad B.T., Wright P.C. (2006). The current status of natural products from marine fungi and their potential as anti-infective agents. J. Ind. Microbiol. Biotechnol..

[12] Motti C.A., Bourne D.G., Burnell J.N., Doyle J.R., Haines D.S., Liptrot C.H., Llewellyn L.E., Ludke S., Muirhead A., Tapiolas D.M. (2007). Screening Marine Fungi for Inhibitors of the C_4_ Plant Enzyme Pyruvate Phosphate Dikinase: Unguinol as a Potential Novel Herbicide Candidate. Appl. Environ. Microbiol..

[13] de Felício R., Pavão G.B., de Oliveira A.L.L., Erbert C., Conti R., Pupo M.T., Furtado N.A.J.C., Ferreira E.G., Costa Lotofu L.V., Young M.C.M. (2015). Antibacterial, antifungal and cytotoxic activities exhibited by endophytic fungi from the Brazilian marine red alga *Bostrychia tenella* (Ceramiales). Brazilian J. Pharmacogn..

[14] Ha T.M., Kim D.-C., Sohn J.H., Yim J.H., Oh H. (2020). Anti-Inflammatory and Protein Tyrosine Phosphatase 1B Inhibitory Metabolites from the Antarctic Marine-Derived Fungal Strain *Penicillium glabrum* SF-7123. Mar. Drugs.

[15] Abdel-Lateff A., König G.M., Fisch K.M., Höller U., Jones P.G., Wright A.D. (2002). New Antioxidant Hydroquinone Derivatives from the Algicolous Marine Fungus *Acremonium* sp.. J. Nat. Prod..

[16] Julianti E., Oh H., Jang K.H., Lee J.K., Lee S.K., Oh D.C., Oh K.-B., Shin J. (2011). Acremostrictin, a highly oxygenated metabolite from the marine fungus *Acremonium strictum*. J. Nat. Prod..

[17] Kim S., Lee C.W., Park S.-Y., Asolkar R.N., Kim H., Kim G.J., Oh S.J., Kim Y., Lee E.-Y., Oh D.-C. (2021). Acremonamide, a Cyclic Pentadepsipeptide with Wound-Healing Properties Isolated from a Marine-Derived Fungus of the Genus *Acremonium*. J. Nat. Prod..

[18] Yang Y., Zhou R., Park S.-Y., Back K., Bae W.K., Kim K.K., Kim H. (2017). 2-Hydroxymelatonin, a Predominant Hydroxylated Melatonin Metabolite in Plants, Shows Antitumor Activity against Human Colorectal Cancer Cells. Molecules.

[19] Ohtani I., Kusumi T., Kashman Y., Kakisawa H. (1991). High-Field FT NMR Application of Mosher’s Method. The Absolute Configurations of Marine Terpenoids. J. Am. Chem. Soc..

[20] Seco J.M., Quiñoá E., Riguera R. (2004). The Assignment of Absolute Configuration by NMR. Chem. Rev..

[21] Nafie L.A., Keiderling T.A., Stephens P.J. (1976). Vibrational Circular Dichroism. J. Am. Chem. Soc..

[22] Hayashi S., Matsuo A., Matsuura T. (1969). Bazzanene, a sesquiterpene hydrocarbon of a new carbon skeleton from *Bazzania pompeana* (Lac.) Mitt. Experientia.

[23] Nagashima F., Momosaki S., Watanabe Y., Takaoka S., Huneck S., Asakawa Y. (1996). Sesquiterpenoids from the liverworts *Bazzania trilobata* and *Porella canariensis*. Phytochemistry.

[24] Asakawa Y., Toyota M., Nagashima F., Hashimoto T. (2008). Chemical Constituents of Selected Japanese and New Zealand Liverworts. Nat. Prod. Commun..

[25] Nagashima F., Toyota M., Asakawa Y. (2006). Bazzanane Sesquiterpenoids from the New Zealand Liverwort *Frullania falciloba*. Chem. Pharm. Bull..

[26] Zhou R., Yang Y., Park S.-Y., Nguyen T.T., Seo Y.-W., Lee K.H., Lee J.H., Kim K.K., Hur J.-S., Kim H. (2017). The lichen secondary metabolite atranorin suppresses lung cancer cell motility and tumorigenesis. Sci. Rep..

[27] Bae W.K., Park M.S., Lee J.H., Hwang J.E., Shim H.J., Cho S.H., Kim D.-E., Ko H.M., Cho C.-S., Park I.-K. (2013). Docetaxel-loaded thermoresponsive conjugated linoleic acid-incorporated poloxamer hydrogel for the suppression of peritoneal metastasis of gastric cancer. Biomaterials.

[28] Nguyen T.T., Yoon S., Yang Y., Lee H.-B., Oh S., Jeong M.-H., Kim J.-J., Yee S.-T., Crişan F., Moon C. (2014). Lichen Secondary Metabolites in *Flavocetraria cucullata* Exhibit Anti-Cancer Effects on Human Cancer Cells through the Induction of Apoptosis and Suppression of Tumorigenic Potentials. PLoS ONE.

[29] Yang Y., Nguyen T.T., Pereira I., Hur J.-S., Kim H. (2019). Lichen Secondary Metabolite Physciosporin Decreases the Stemness Potential of Colorectal Cancer Cells. Biomolecules.

[30] Zhou R., Yang Y., Park S.Y., Seo Y.W., Jung S.C., Kim K.K., Kim K., Kim H. (2019). p300/CBP-associated factor promotes autophagic degradation of δ-catenin through acetylation and decreases prostate cancer tumorigenicity. Sci. Rep..

[31] Taş I., Han J., Park S.-Y., Yang Y., Zhou R., Gamage C.D.B., Van Nguyen T., Lee J.-Y., Choi Y.J., Yu Y.H. (2018). Physciosporin suppresses the proliferation, motility and tumourigenesis of colorectal cancer cells. Phytomedicine.

