# Supplementary material for: Marinobazzanan, a Bazzanane-Type Sesquiterpenoid, Suppresses the Cell Motility and Tumorigenesis in Cancer Cells
Source: Mar Drugs. 2023 Feb 25;21(3):153. doi: 10.3390/md21030153 (PMC10056982; doi:10.3390/md21030153)

Supporting Information

Marinobazzanan, a Bazzanane-Type Sesquiterpenoid, Suppresses the Cell Motility and Tumorigenesis in Cancer Cell

Sultan Pulat [1,†], Prima F. Hillman [2,†], Sojeong Kim [3], Ratnakar N. Asolkar [4], Haerin Kim [3], Rui Zhou [1], İsa Taş [1], Chathurika D.B. Gamage [1], Mucahit Varli [1], So-Yeon Park [1], Sung Chul Park [5], Inho Yang [6], Jongheon Shin [5], Dong-Chan Oh [5], Hangun Kim [1,], Sang-Jip Nam [2,*], and William Fenical [4,*]*

[1]College of Pharmacy and Research Institute of Life and Pharmaceutical Sciences, Sunchon National University, Sunchon 57922, Republic of Korea

[2] Department of Chemistry and Nanoscience, Ewha Womans University, Seoul 03760, Republic of Korea

[3] Graduate School of Industrial Pharmaceutical Sciences, Ewha Womans University, Seoul 03760, Republic of Korea

[4]Center for Marine Biotechnology and Biomedicine, Scripps Institution of Oceanography, University of California-San Diego, La Jolla, CA 92093-0204, USA

[5]Natural Products Research Institute, College of Pharmacy, Seoul National University, San 56-1, Sillim, Gwanak, Seoul 08826, Republic of Korea

[6]Department of Convergence Study on the Ocean Science and Technology, Korea Maritime and Ocean University, Busan 49112, Republic of Korea

*Correspondence: hangunkim@scnu.ac.kr (H.K.); sjnam@ewha.ac.kr (S.-J.N.); wfenical@ucsd.edu (W.F.)

† These authors contributed equally to this work.

Table of Contents

Figure S1. ^{1}H NMR spectrum (400 MHz) of marinobazzanan (**1**) in CD_3OD. **S3**

Figure S2. ^{13}C NMR spectrum (75 MHz) of marinobazzanan (**1**) in CD_3OD. **S4**

Figure S3. COSY NMR spectrum (500 MHz) of marinobazzanan (**1**) in CD_3OD. **S5**

Figure S4. HSQC NMR spectrum (500 MHz) of marinobazzanan (**1**) in CD_3OD. **S6**

Figure S5. HMBC NMR spectrum (500 MHz) of marinobazzanan (**1**) in CD_3OD. **S7**

Figure S6. NOESY NMR spectrum (500 MHz) of marinobazzanan (**1**) in CD_3OD. **S8**

Figure S7. ^{1}H NMR spectrum (500 MHz) of *S*-MTPA ester (**1a**) for marinobazzanan (**1**) in $CDCl_3$. **S9**

Figure S8. ^{1}H NMR spectrum (500 MHz) of *R*-MTPA ester (**1b**) for marinobazzanan (**1**) in $CDCl_3$. **S10**

Figure S9. The effect of various fraction of marinobazzanan (**1**) upon the cell viability of Caco2. .... **S11**

Figure S10. LRMS spectrum of marinobazzanan (**1**). **S12**

Figure S1. ^{1}H NMR spectrum (500 MHz) of marinobazzanan (**1**) in CD_3OD.

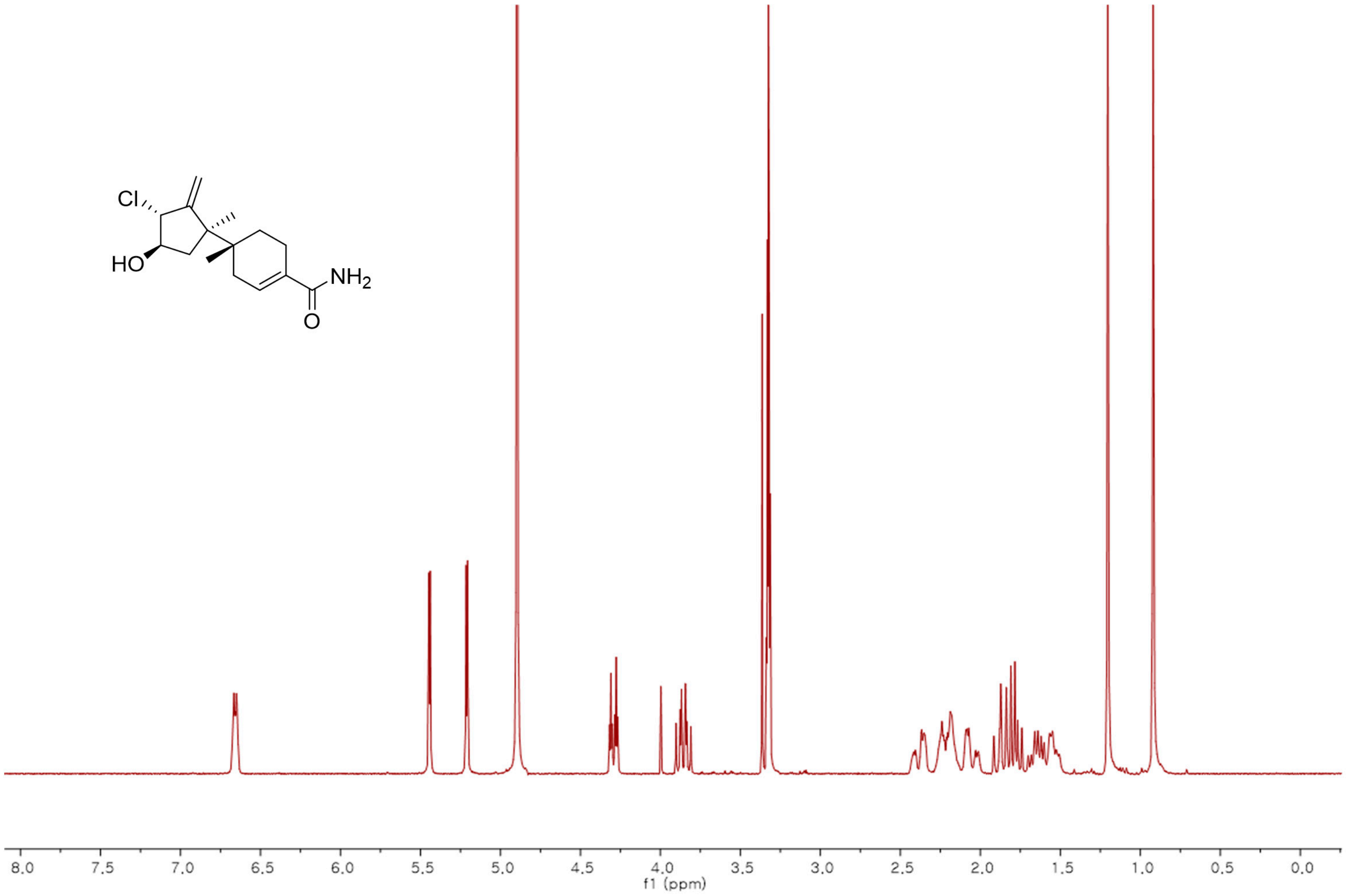

Figure S2. ^{13}C NMR spectrum (75 MHz) of marinobazzanan (**1**) in CD_3OD.

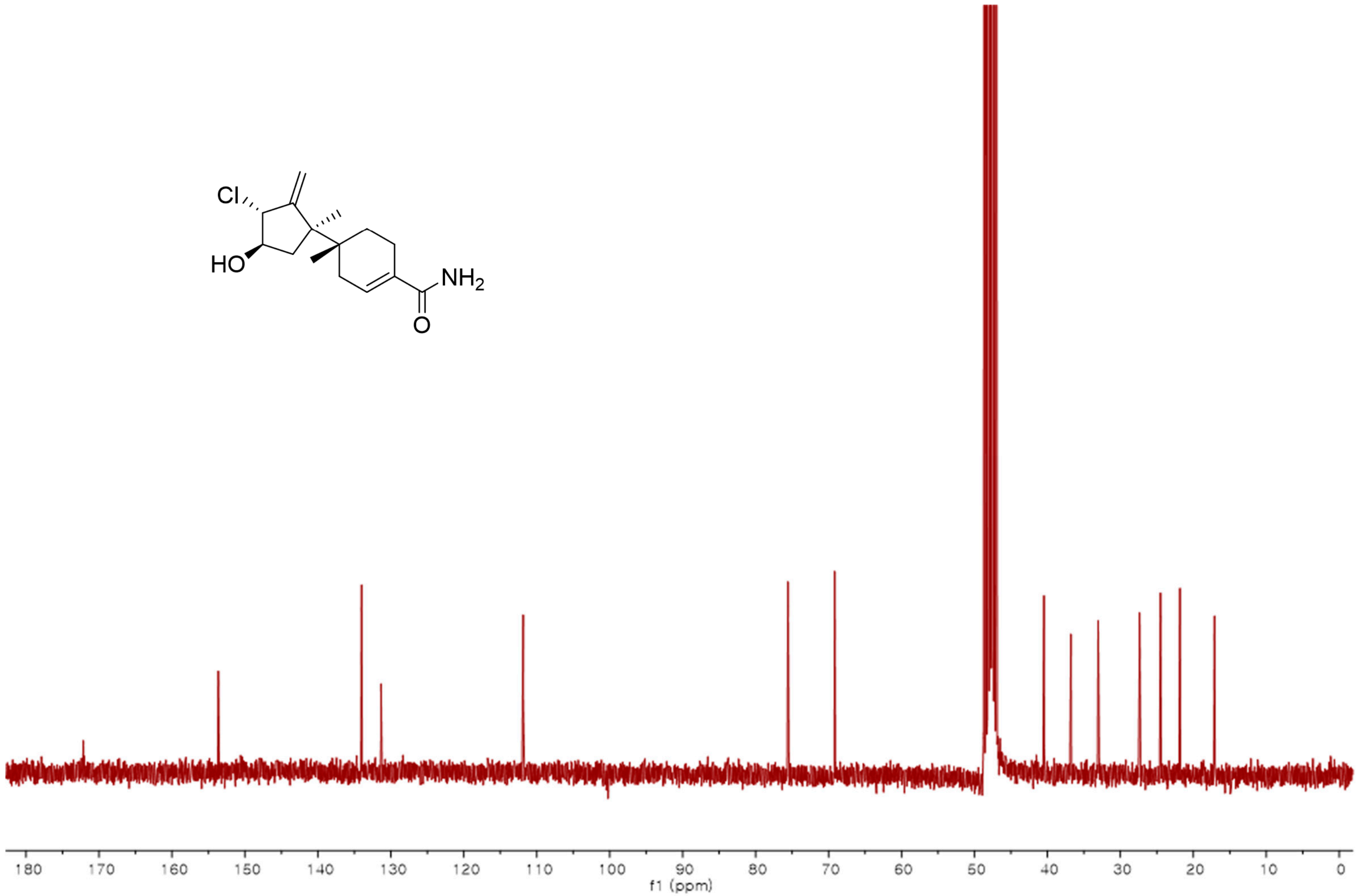

Figure S3. COSY NMR spectrum (500 MHz) of marinobazzanan (**1**) in CD_3OD.

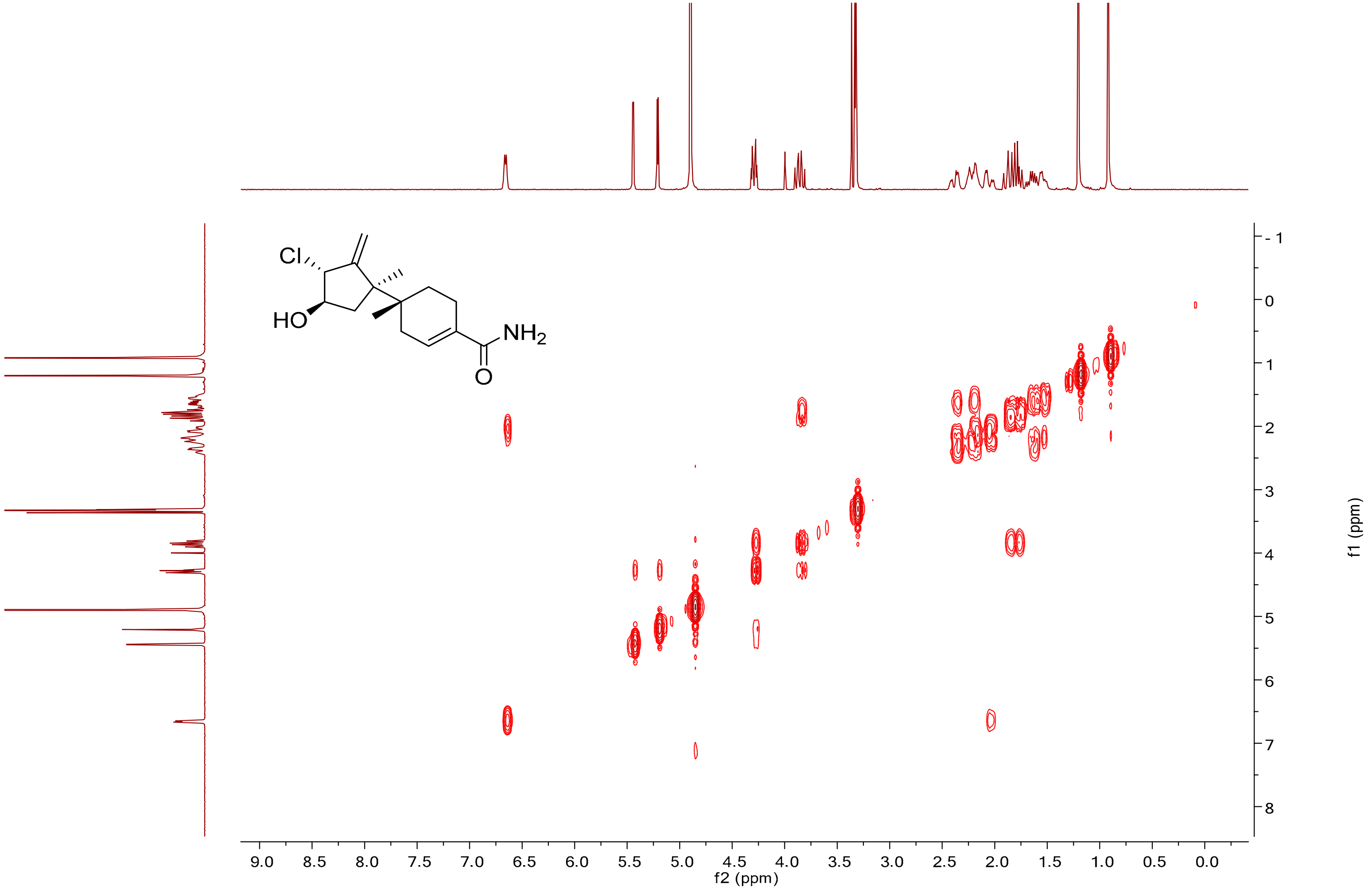

Figure S4. HSQC NMR spectrum (500 MHz) of marinobazzanan (**1**) in CD_3OD.

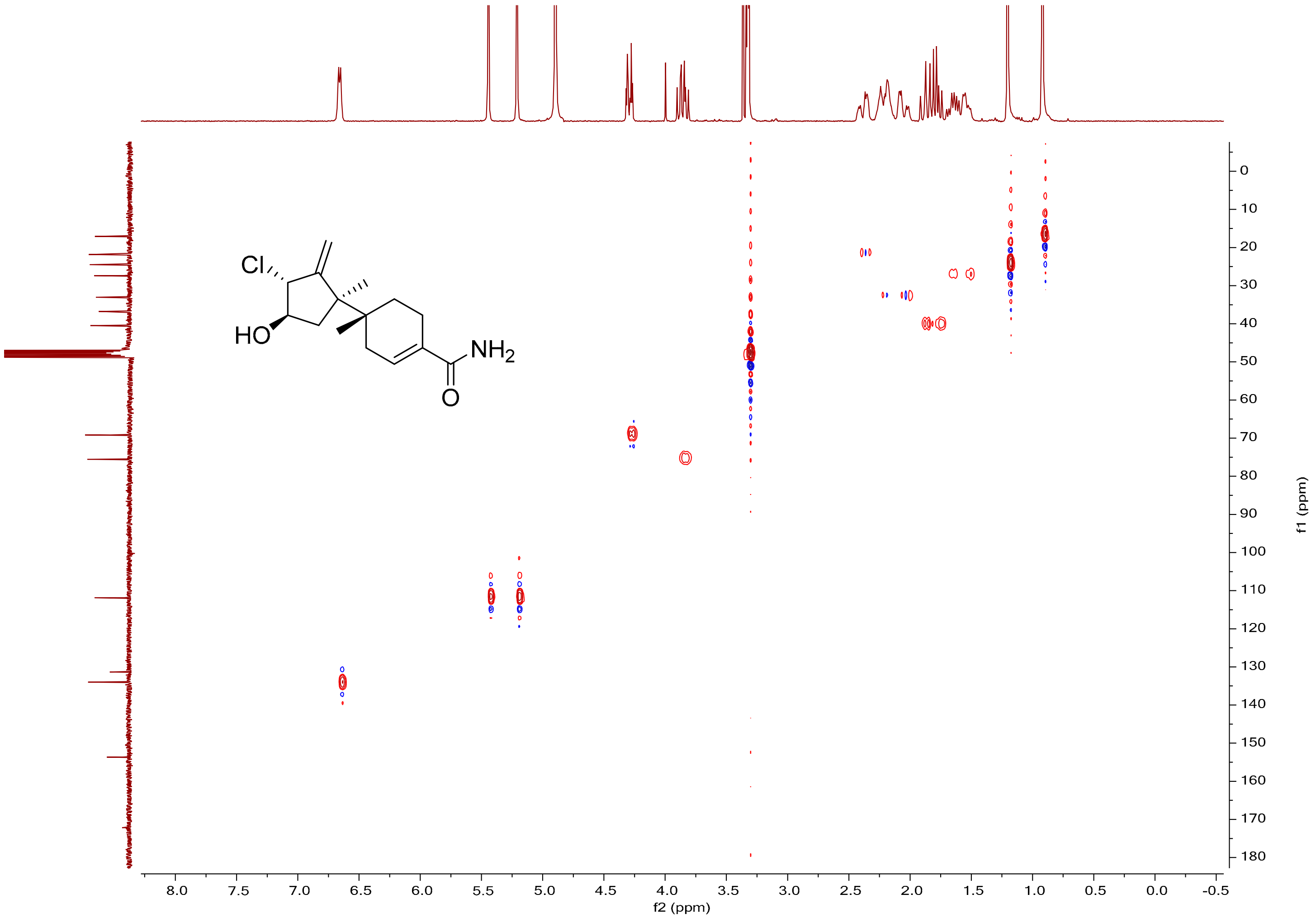

Figure S5. HMBC NMR spectrum (500 MHz) of marinobazzanan (**1**) in CD_3OD.

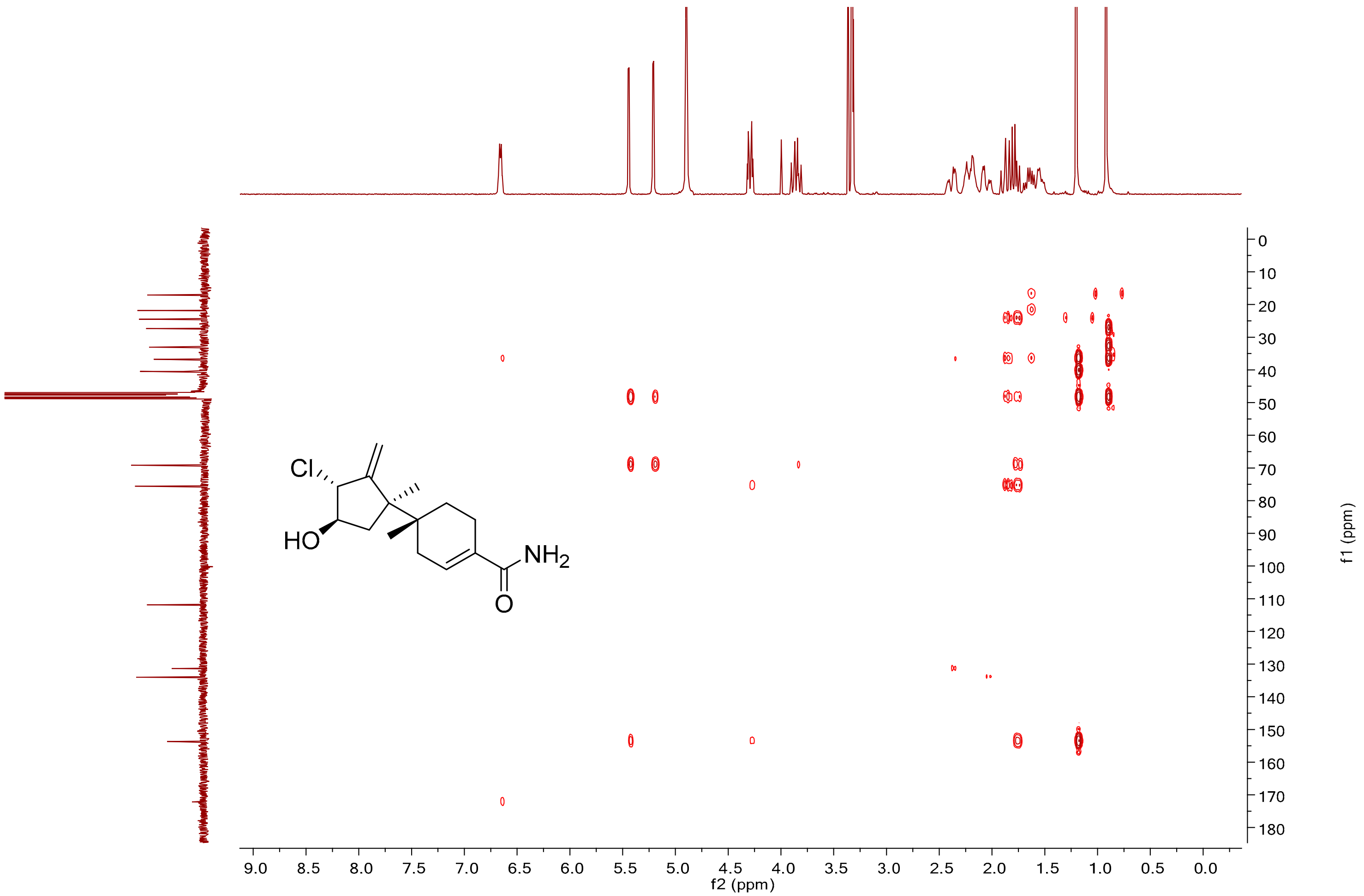

Figure S6. NOESY NMR spectrum (500 MHz) of marinobazzanan (**1**) in CD_3OD.

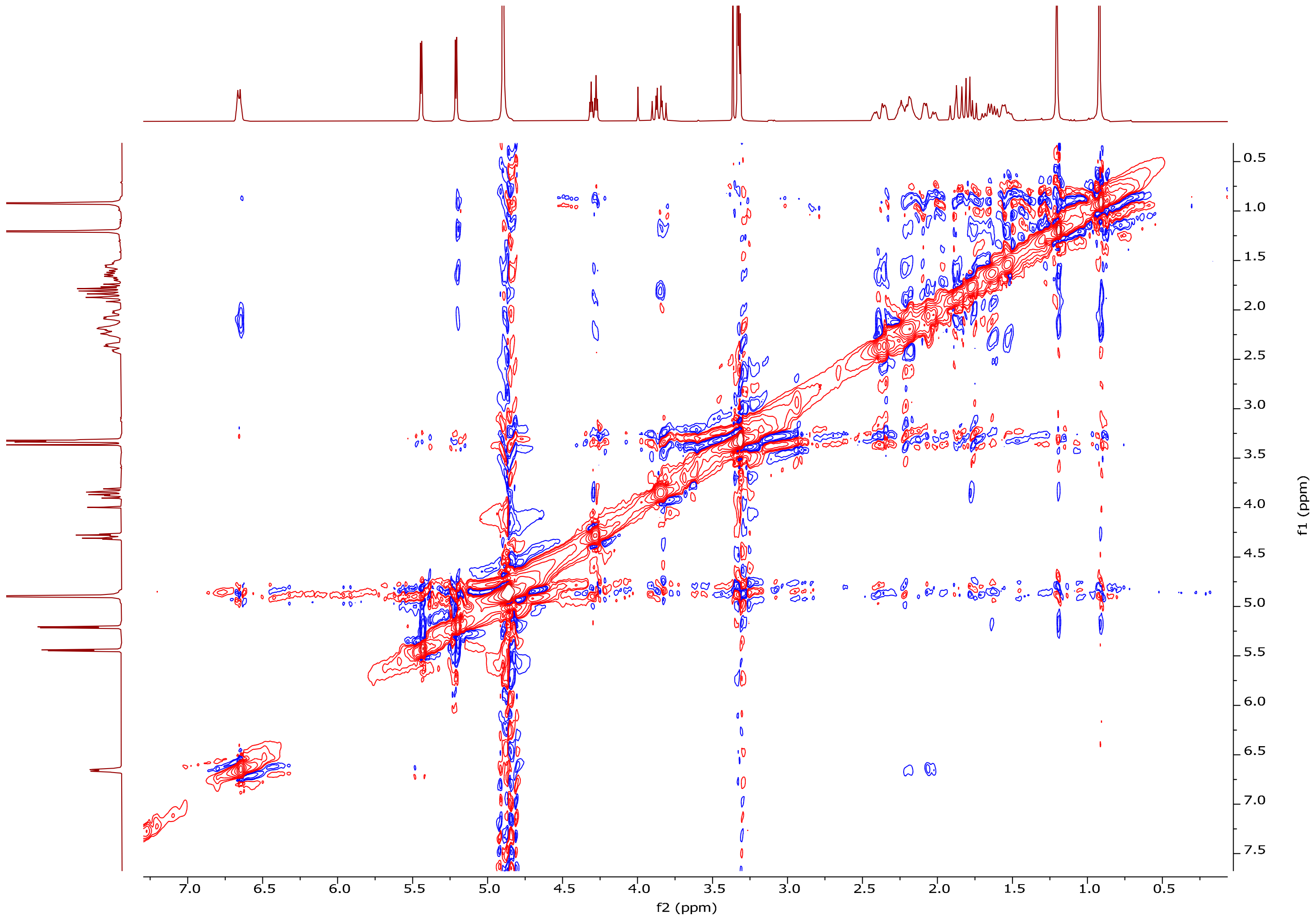

Figure S7. ¹H NMR spectrum (500 MHz) of *S*-MTPA ester (**1a**) for marinobazzanan (**1**) in $CDCl_3$.

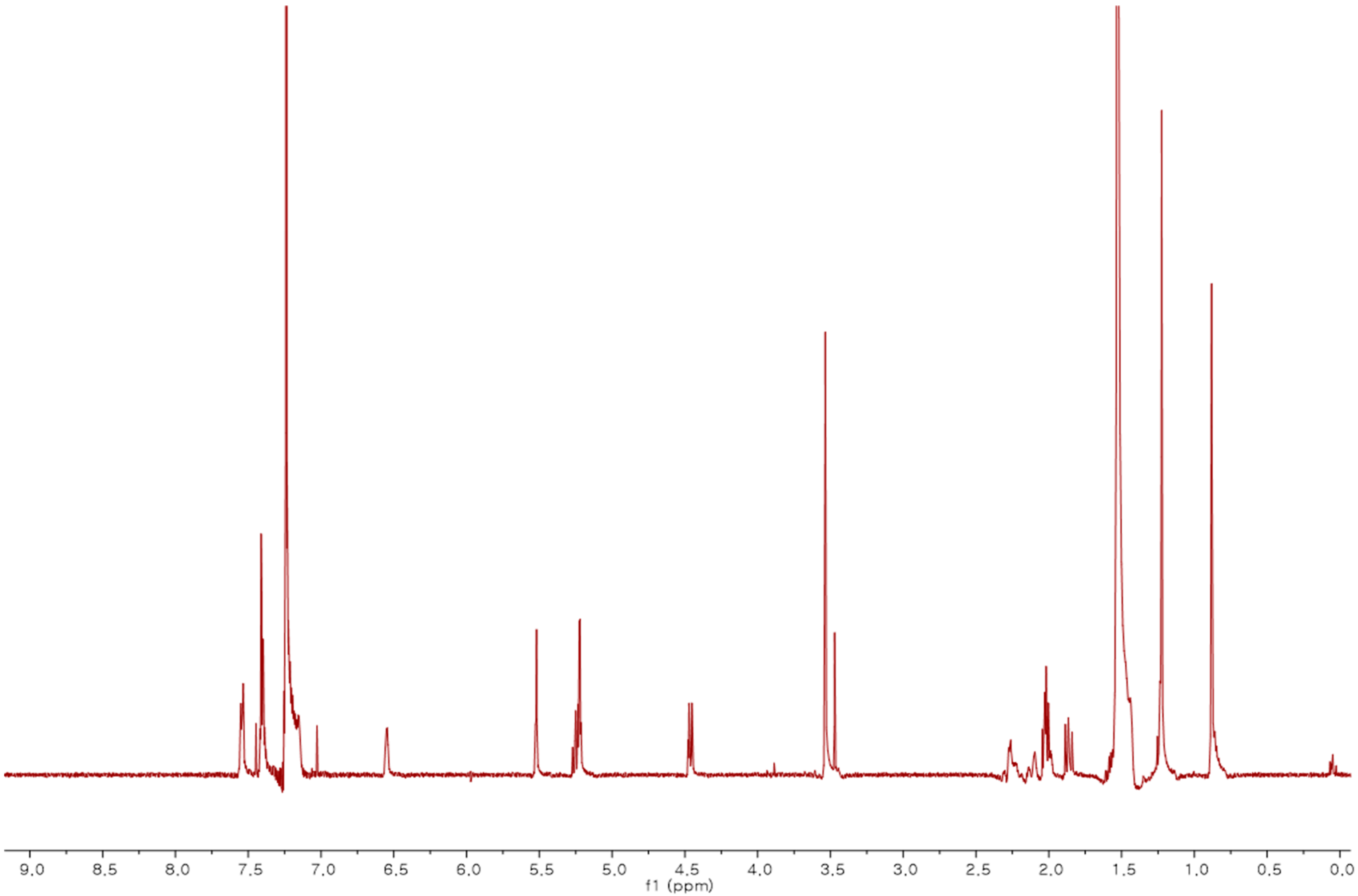

Figure S8. ^{1}H NMR spectrum (500 MHz) of *R*-MTPA ester (**1b**) for marinobazzanan (**1**) in $CDCl_3$.

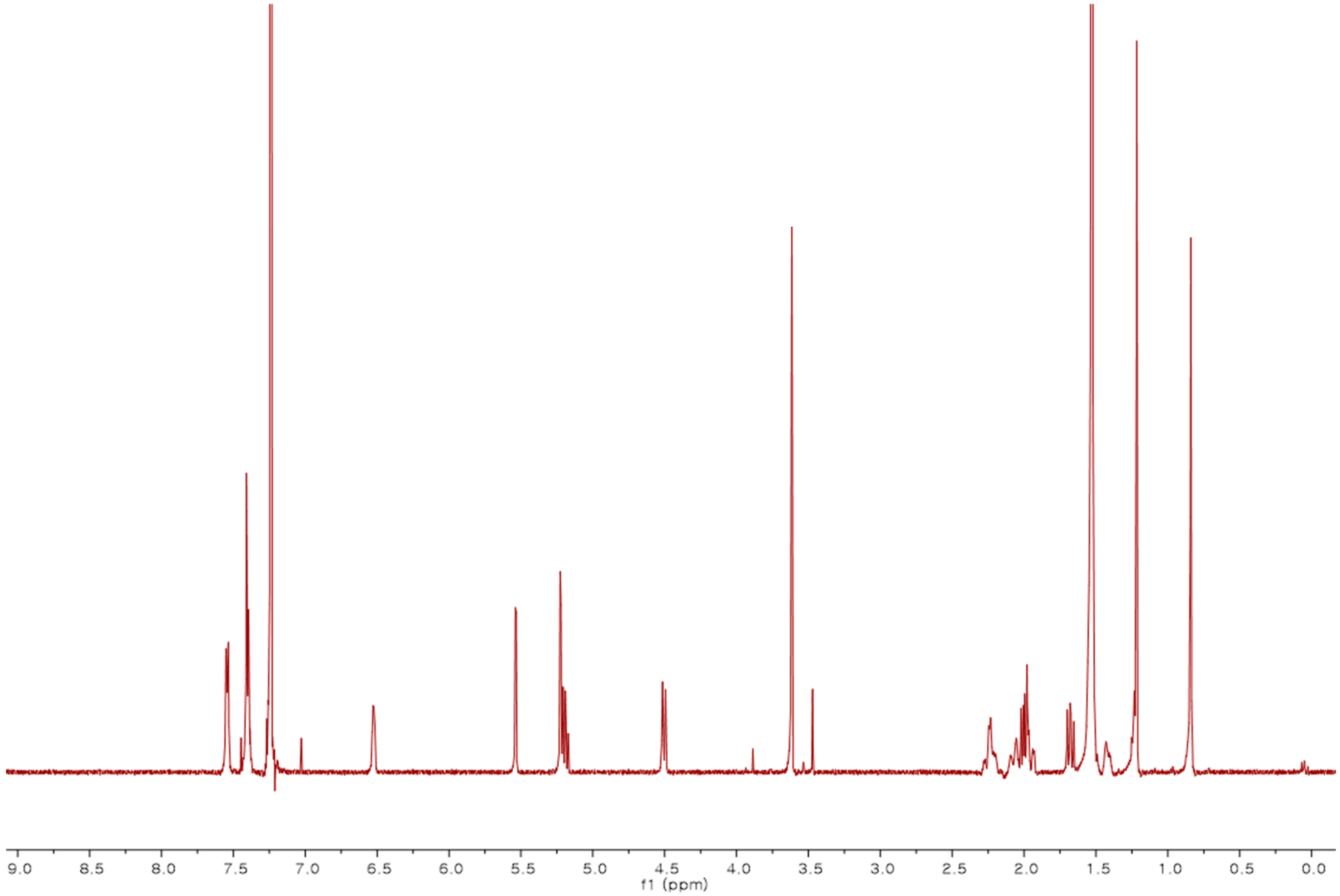

Figure S9. The effect of various fraction of marinobazzanan (**1**) upon the cell viability of Caco2.

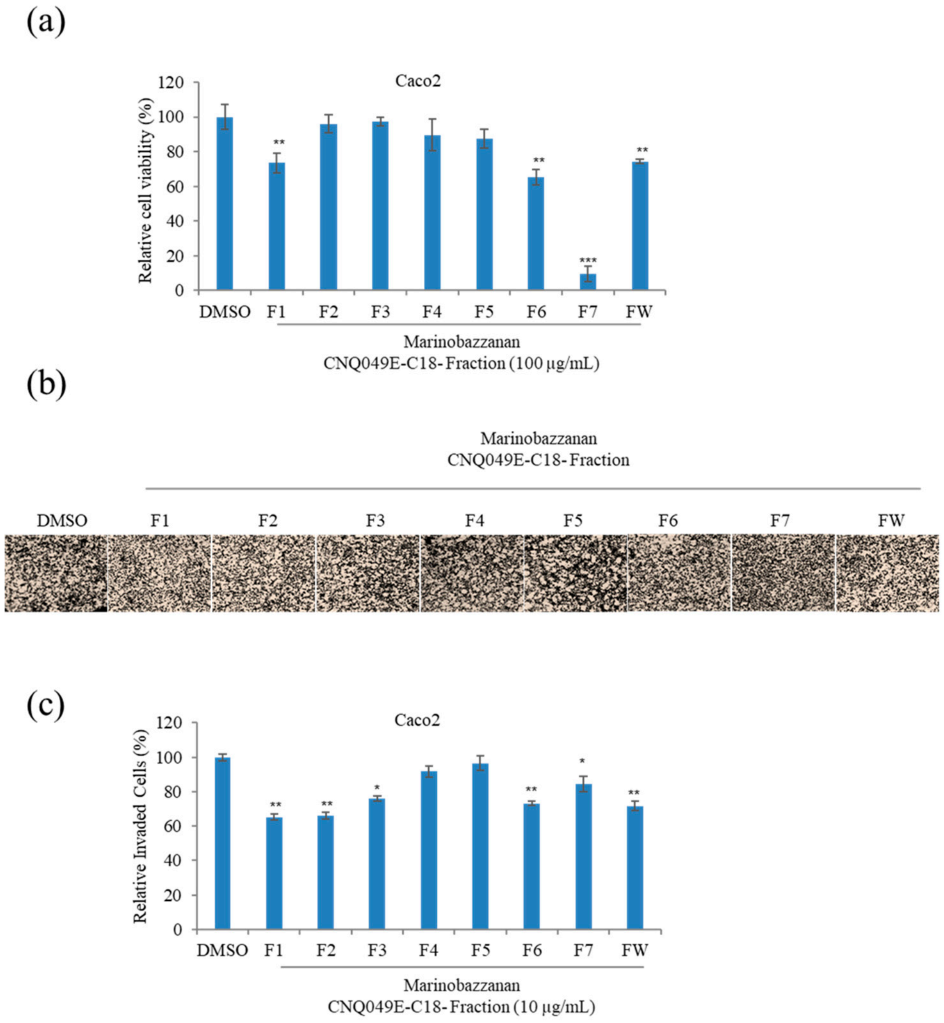

Figure S10. LRMS spectrum of marinobazzanan (**1**).

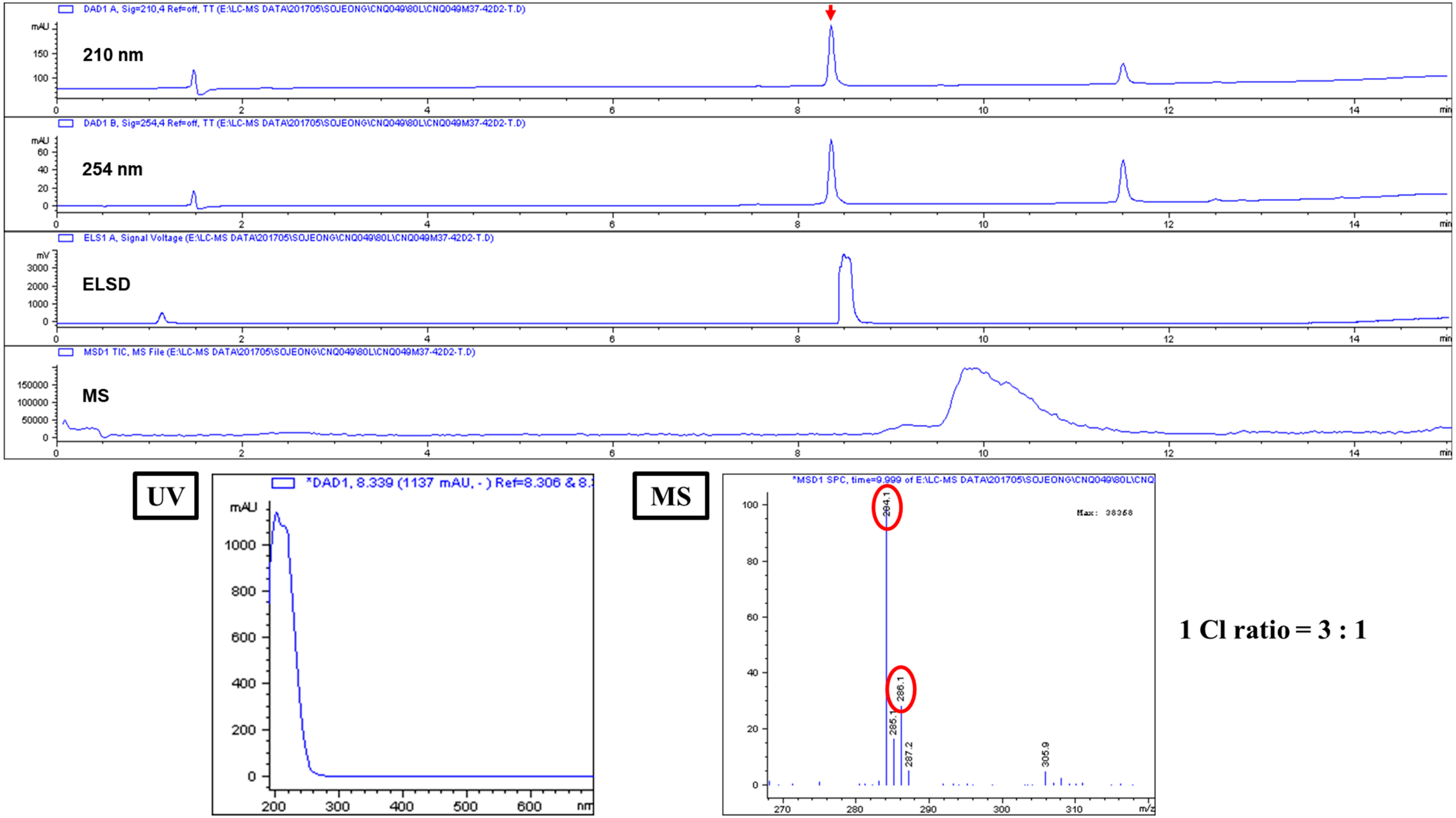

Supplement: Supplementary file 1 [file marinedrugs-21-00153-s001.zip › marinedrugs-2224265-supplementary.pdf]
